# Metagenomic analysis of the DNA virome communities in swine lungs

**DOI:** 10.3389/fmicb.2026.1798033

**Published:** 2026-05-28

**Authors:** Zifeng Liu, Yiwen Xiahou, Jingquan Li, Fusheng Wu, Yuhang Fan, Ruirong Liu, Mingfang Zhou, Zifeng Ding, Yang Zhang, Congying Chen, Lusheng Huang, Huashui Ai

**Affiliations:** National Key Laboratory for Swine Genetic Improvement and Germplasm Innovation, Jiangxi Agricultural University, Nanchang, China

**Keywords:** auxiliary metabolic genes, DNA virome, domestic pig, lung, viral operational taxonomic units, wild boar

## Abstract

Viruses play critical roles in shaping microbial communities and regulating host metabolism. Investigating the lung virome of pigs can inform swine health management and provide a comparative resource for studies of the human lower respiratory virome. However, viral communities in the porcine lower respiratory tract remain poorly characterized. In this study, lung-associated viral communities were investigated using virus-like particle (VLP) enrichment and DNA metagenomic sequencing of 49 lung-derived samples collected from 17 domestic pigs and 20 wild boars. A total of 18,412 viral operational taxonomic units (vOTUs) were identified. Among the 2,559 vOTUs with genome completeness ≥50%, nearly 95% did not cluster with sequences in current viral reference databases at the species-level threshold (ANI ≥ 95% and AF ≥ 85%), suggesting putative viral novelty in the porcine lung while also reflecting incomplete reference database coverage. Meanwhile, 10,819 vOTUs (accounting for 58.8% of the total 18,412 identified vOTUs) were assigned to known viral taxa, spanning 29 viral orders and 65 viral families. The most prevalent viral families were *Microviridae*, *Circoviridae*, *Smacoviridae*, *Adintoviridae*, and *Autographiviridae*. Host prediction linked a subset of vOTUs to putative bacterial hosts, mainly from Pseudomonadota, Bacillota, Bacteroidota and Actinomycetota. In addition, we identified 191 vOTUs carrying 40 auxiliary metabolic genes (AMGs) mapped to 31 metabolic pathways. These AMGs were mainly associated with sulfur metabolism, cysteine and methionine metabolism, folate biosynthesis, and one-carbon pool by folate pathways. Comparative analysis under this study design showed that domestic pigs harbored higher viral diversity with a greater number of unique vOTUs (*n* = 12,611) than wild boars (*n* = 3,072). Domestic pigs viromes were enriched in *Circoviridae* and *Microviridae,* whereas wild boars showed higher relative abundances of *Adintoviridae* and *Genomoviridae*. Putative AMGs related to coenzyme synthesis and DNA methylation were more frequently detected in domestic pigs, whereas AMGs associated with nucleotide biosynthesis and cofactor metabolism were enriched in wild boars. These findings characterize the composition and functional potential of lung-associated DNA viral communities in pigs and provide a resource for future respiratory virome studies.

## 1 Introduction

Viruses are ubiquitously distributed across diverse ecosystems, ranging from water, glaciers, and soil to host-associated niches such as the gut, respiratory tract, and reproductive system (Paez-Espino et al., 2016; Suttle, 2007). The term virome refers specifically to the collection of viruses within environment or host (Santiago-Rodriguez and Hollister, 2022). As one of the most abundant and genetically diverse biological entities on Earth, viruses play important roles in shaping microbial community structure and influencing host-associated ecological processes (Breitbart and Rohwer, 2005; Barton et al., 2007). Previous studies have suggested that viruses may regulate microbial populations through mechanisms such as the “kill-the-winner” and “piggyback-the-winner” hypotheses (Thingstad, 2000; Silveira and Rohwer, 2016). In addition, viruses may carry auxiliary metabolic genes (AMGs) that influence host metabolism and contribute to the community-level nutrient cycling (Hurwitz and U’Ren, 2016; Breitbart et al., 2018).

Within mammalian hosts, the virome actively influences the abundance and functional characteristics of bacteria at barrier surfaces (Virgin, 2014). Meanwhile, persistently present eukaryotic ssDNA viruses, particularly *Anelloviridae* and CRESS-DNA (Circular Rep-encoding ssDNA) viruses, interact with host immune responses and have been proposed as potential indicators of mucosal immune status (Kaczorowska and Van Der Hoek, 2020; Abbas et al., 2019; Young et al., 2015). The respiratory tract is a distinct biological environment, where continuous exposure to inhaled particles, mucociliary clearance, surfactant mediated barrier functions, and airway specific immune defenses together impose selective pressures on viral persistence. In addition, the relatively low microbial biomass in the lower airways may enhance stochastic processes during community assembly (Natalini et al., 2023; Dickson et al., 2016). Together, host immune responses and local microbial communities may make the lower airway virome distinct from viromes at other body sites.

Metagenomic studies have begun to characterize the respiratory virome in humans under both healthy and diseased conditions (Willner et al., 2009; Zhang et al., 2023). These studies suggest that the respiratory tract may harbor recurrently detected eukaryotic DNA viruses and bacteriophages in healthy individuals, and viral communities can shift in disease (Willner et al., 2009; Conradie et al., 2024). Three factors still limit our understanding of lung-associated viromes. First, the low viral biomass in the lower respiratory tract restricts genome recovery and reduces analytical resolution. Second, many viral sequences show little similarity to existing reference databases, which makes taxonomic and functional annotation challenging (Nayfach et al., 2021). Third, most current studies focus on human populations, while comparable data from other mammals remain limited (Carroll et al., 2018). As a result, the diversity, host associations, and functional roles of lung viromes remain poorly understood, particularly in non-human hosts.

Swine, as a valuable biomedical model, is well suited for studying the respiratory virome. Their lower respiratory tract is similar to that of humans in several anatomical and physiological features, including lung structure, mucociliary clearance, and surfactant composition (Rogers et al., 2008; Judge et al., 2014; Meyerholz et al., 2018). They have immune systems more similar to those of humans than rodent models, supporting their use in studies of host virus interactions in the airway (Meurens et al., 2012). In addition, they are central to zoonotic virus ecology, supporting replication of both avian and mammalian influenza viruses and facilitating cross species transmission (Nelson and Vincent, 2015). Close contact with humans in agricultural and transport increases the likelihood of viral exchange (Borkenhagen et al., 2019; Reperant et al., 2024). These features support the use of swine for investigating mammalian lung viromes and help improve our understanding of their diversity and function.

In this study, we used metagenomic approaches to investigate the DNA virome of the lower respiratory tract in domestic pigs and wild boars raised under natural conditions. The main aim was to characterize the diversity and composition of lung associated viral communities. To address this, we assessed the extent of viral diversity and phylogeny, compared community composition and functional potential between domestic pigs and wild boars, and investigated potential virus host interactions and functional features. These analyses provide a framework for understanding lung viromes in mammals and offer new insights into their diversity and potential ecological roles.

## 2 Materials and methods

### 2.1 Experimental animals

A total of 37 pigs were included in this study, comprising 17 domestic pigs and 20 wild boars. Among the domestic pigs, 11 were commercial pigs of European origin, and six belonged to a mosaic pig population. The mosaic population was developed by crossing four Western breeds (Duroc, Landrace, Large White, and Pietrain) with four Chinese indigenous breeds (Bamaxiang, Erhualian, Laiwu, and Tibetan) using a disc rotation breeding system, as previously described (Yang et al., 2022). All mosaic pigs were raised under standardized indoor conditions at an experimental farm in Ganzhou, Jiangxi Province, China. The 11 commercial pigs were European hybrid pigs obtained from a local market. Wild boars were captured from mountainous regions across Liaoning, Hubei, Hunan, Shaanxi, and Ningxia provinces in China (Supplementary Figure S1A). This design provides a descriptive comparison between wild boars from natural environments and domestic pigs raised under standardized farm conditions.

### 2.2 Sample collection

A total of 26 bronchoalveolar lavage (BAL) samples were collected from 20 wild boars and six mosaic pigs. The 11 commercial pigs were analyzed separately and divided into two groups. In the first group (six pigs), BAL samples were collected and each sample was split into two aliquots. One aliquot was sequentially filtered through 0.45 μm and 0.22 μm membranes to enrich virus-like particles (VLPs), whereas the other aliquot was filtered through 0.45 μm and 0.10 μm membranes, yielding a total of 12 BAL-derived samples. In the second group (five pigs), a total of 11 samples were collected, including three BAL samples, four lung tissue expression (LTE) samples, and four lung tissue homogenate (LTH) samples.

For BAL samples, lungs were separated immediately after slaughter and lavaged with sterile phosphate-buffered saline (PBS). Wild boars were slaughtered at the capture site and transported to the sampling location within approximately 30 min prior to lung processing. The lungs were then gently kneaded and squeezed for approximately 2 min before the lavage fluid was recovered. Approximately 50 mL of bronchoalveolar lavage fluid was collected from each pig within 30 min, avoiding contact with external materials. For LTE and LTH samples, a 2 × 1 × 1 cm section of lung tissue was excised from the same anatomical region and placed into a 50 mL tube containing sterile PBS. For LTE samples, the tissue was manually dissected and squeezed back and forth for approximately 2 min, and the resulting eluate (approximately 20 mL) was collected. For LTH samples, lung tissue was homogenized in a 50 mL tube for 2 min using a homogenizer, and approximately 20 mL of the resulting homogenate was collected. All samples were centrifuged at 4,000 × g for 30 min at 4 °C (Esco Life Sciences, Singapore). The resulting supernatant was adjusted to a final volume of 50 mL with sterile PBS and either processed immediately or stored at −80 °C until further use. Comparative analyses of these methodological variables are presented in Section 3.3.

### 2.3 VLP enrichment

A total of 25 mL of supernatant from each sample was sequentially filtered through 0.45 μm and 0.22 μm (or 0.10 μm) membrane filters (Merck Millipore, Ireland) to remove bacterial contaminants, and the filtrate was collected into a sterile 50 mL centrifuge tube. For supernatant stored at −80 °C, samples were first thawed and vortexed for 10 s prior to filtration, and 25 mL of the liquid was transferred for processing. The filtrate was subsequently concentrated using Amicon Ultra-15 Filter Units (Merck Millipore, Ireland) at 5,000 × g for 30 min at 4 °C until a final volume of approximately 500 μL was obtained. The concentrate was resuspended in 1 mL of SM buffer (50 mM Tris–HCl at pH 7.5, 100 mM NaCl, 8 mM MgSO4, 0.01% gelatin), transferred to a 15 mL tube, and treated with lysozyme (0.01 volumes; Thermo Fisher Scientific, USA) at 37 °C for 1 h to further reduce bacterial contamination. Chloroform (0.2 volumes) was then added, and the mixture was incubated at room temperature for 10 min, this step may reduce recovery of lipid-containing enveloped viruses. The resulting supernatant was transferred to a 2 mL Eppendorf tube and allowed to stand at room temperature for 5 min. To remove free nucleic acids, 6 μL of DNase reaction buffer (Lucigen, USA) and 3 μL of Baseline-ZERO DNase (Lucigen, USA) were added, followed by incubation at 37 °C for 60 min. The reaction was terminated by adding 6 μL of stop buffer (Lucigen, USA) and heating at 65 °C for 10 min, yielding the final VLP preparation.

Negative controls (*n* = 4) were processed in parallel by filling 50 mL centrifuge tubes with sterile PBS from the same batch and subjecting them to identical procedures as the experimental samples.

### 2.4 DNA extraction and sequencing

Viral DNA was extracted from the final VLP preparation using the ZR Viral DNA/RNA Kit (Zymo Research, USA) according to the manufacturer’s instructions. All extraction procedures were conducted aseptically in a biosafety cabinet. Whole-genome amplification was performed using the illustra Ready-To-Go GenomiPhi V3 DNA Amplification Kit (Cytiva, USA) following the manufacturer’s instructions. Multiple Displacement Amplification (MDA) may preferentially amplify circular DNA templates and affect relative abundance estimates. The same protocol was applied to all samples to reduce potential group specific bias. The quality of amplified DNA products was assessed using a NanoDrop One spectrophotometer (Thermo Fisher Scientific, USA), a Qubit 4.0 fluorometer (Life Technologies, USA), and 1.5% agarose gel electrophoresis. Viral metagenomic libraries were constructed using the ALFA-SEQ DNA Library Prep Kit DL1010 (Guangzhou mCHIP Biotech Co., Ltd., Guangzhou, China) following the manufacturer’s protocol. Library quality was evaluated using a Qubit 4.0 fluorometer (Life Technologies, USA) and an Agilent 4,200 TapeStation (Agilent Technologies, USA). After qualification and purification, libraries were pooled into the flow cell according to effective concentration and target sequencing depth. Paired-end sequencing (150 bp) was performed on the DNBSEQ-T7 platform (BGI, Shenzhen, China).

### 2.5 Quality control, assembly, and viral identification

Raw sequencing reads were trimmed and quality filtered using fastp v0.23.4 (Chen et al., 2018). Host-derived reads were removed by aligning filtered reads against the *Sus scrofa* reference genome (Sscrofa11.1) using Bowtie2 v2.5.4 (Langmead and Salzberg, 2012). *De novo* assembly of host-filtered reads was performed separately for each sample using MEGAHIT v1.2.9 (Li et al., 2014). Contigs with a minimum length of 1.5 kb were retained for downstream viral identification. Putative viral contigs were identified using VirSorter2 v2.2.4 (Guo et al., 2021), DeepVirFinder v1.0 (Ren et al., 2020), and VIBRANT v1.2.1 (Kieft et al., 2020), all run with default parameters.

A contig was classified as viral if it met at least one of the following criteria: (1) identified by VirSorter2 with a score ≥ 0.8; (2) identified by DeepVirFinder with a score ≥ 0.8 and *p* < 0.05; or (3) identified by VIBRANT. For contigs detected by VirSorter2, corresponding VIBRANT predictions were filtered based on classification type (“full,” “lt2gene,” or “partial”). Contigs classified by VirSorter2 as “full” or “lt2gene” were retained solely based on VirSorter2 classifications. For VirSorter2 predictions classified as “partial” that matched a VIBRANT “fragment” classification, both results were retained. All viral contigs identified by the above approaches were integrated into a non-redundant viral sequence dataset using a custom script. Finally, plasmid sequences were removed using geNomad v1.7.4 (Camargo et al., 2023), whose plasmid classifier covers bacterial plasmids (PLSDB, release 2020_11_19) (Schmartz et al., 2022), archaeal plasmids (RefSeq, retrieved 23 July 2021), and metagenomic plasmid-like sequences (IMG/M ID: 3300053491), to yield the final viral sequence dataset for downstream analyses, representing putative viral sequences. This multi-tool identification approach was used to reduce false-positive viral identification.

### 2.6 Quality assessment and viral contig clustering

The quality and completeness of viral sequences were initially assessed using CheckV v1.0.1 with the accompanying database v1.5 (Nayfach et al., 2020). Viral contigs were clustered into species-level viral operational taxonomic units (vOTUs) based on thresholds of 95% average nucleotide identity (ANI) and 85% alignment fraction (AF). Pairwise ANI calculation followed the rapid genome clustering support scripts provided in the CheckV repository. All-versus-all local sequence alignments were performed using BLAST+ v2.14.1 (Camacho et al., 2009). Pairwise ANI values were calculated by integrating local alignments between sequence pairs using the anicalc.py script. Based on MIUVIG’s (Roux et al., 2019) recommended thresholds of 95% ANI and 85% AF, UCLUST-like clustering was performed using the aniclust.py script to define vOTUs. Following clustering, representative vOTUs sequences were extracted, and CheckV was re-applied to re-evaluate the quality and completeness of each representative vOTU. vOTUs with genome completeness ≥50% were grouped into three categories following MIUVIG criteria: Complete (100% completeness), High-quality (≥90%), and Medium-quality (50–90%).

### 2.7 Viral taxonomic identification and abundance calculation

Taxonomic classification of vOTUs was performed using two methods based on the International Committee on Taxonomy of Viruses (ICTV): geNomad and PhaBox v2.0.1 (Shang et al., 2023). When both tools provided annotations for the same vOTU, classifications from geNomad were prioritized. Information on the predicted genetic code of each vOTU was obtained from geNomad and used in downstream analyses. Host-filtered reads were mapped to vOTUs using BWA-MEM v0.7.17 (Li and Durbin, 2009), and the resulting SAM files were converted into BAM format using SAMtools. Relative abundance of vOTUs per sample was calculated using CoverM v0.6.1 (Aroney et al., 2025) in contig mode and expressed as the reads per kilobase per million mapped reads (RPKM). Additionally, abundance calculations were performed separately for vOTUs with genome completeness of at least 50%, as determined by CheckV.

To reduce the influence of low-abundance signals, abundance-based filtering criteria were applied for downstream analyses. vOTUs detected in only a single sample and those with extremely low relative abundance were excluded based on predefined RPKM thresholds.

### 2.8 Functional annotation and putative AMGs

All protein-coding genes of each vOTU were predicted using Prodigal v2.6.3 (Hyatt et al., 2010). Functional annotation was performed against the Structured Antibiotic Resistance Gene database (SARG), the Mobile Genetic Elements database (MGEs), the antimicrobial and metal resistance genes database (BacMet), the Virulence Factor Database (VFDB), and the Carbohydrate-Active enZyme database (CAZy). Protein sequences were aligned against the BacMet, VFDB, CAZy, and MGEs databases using DIAMOND BLASTp with the parameters “*E*-value ≤ 1 × 10^−5^ and max-target-seqs = 1”. Putative auxiliary metabolic genes (AMGs) were identified using VIBRANT with default parameters across all vOTUs and separately vOTUs with genome completeness of at least 50%. The abundance of AMGs was quantified using CoverM with the same parameters as described above.

### 2.9 Host prediction and lifestyle prediction

Virus-host linkages were predicted using iPHoP v1.3.3 (Roux et al., 2023) with the Aug_2023_pub_rw database and default parameters, applying a false discovery rate (FDR) threshold < 10%. iPHoP integrates a phage-based tool (RaFAH) and multiple host-based tools, including BLAST, CRISPR, VirHostMatcher, WIsH, and PHP, to infer host taxonomy, primarily at the genus level. When multiple host predictions were obtained for a given vOTU, only the prediction with the highest confidence score was retained. Viral lifestyle prediction was performed using DeePhage v1.0 (Wu et al., 2021). The DeePhage virtual machine image was obtained from the Zhu Lab website and executed using VirtualBox v7.1.6. According to DeePhage classification scores, vOTUs were classified to one of four lifestyle categories: temperate (score ≤ 0.3), uncertain temperate (0.3 < score ≤ 0.5), uncertain virulent (0.5 < score ≤ 0.7), and virulent (score > 0.7) (Lu et al., 2024; Chen et al., 2024).

### 2.10 Comparison to the viral reference databases

In this study, vOTUs from the swine lung DNA virome were compared against five public databases, including the Metagenomic Gut Virus Catalogue (MGV), the Viral Reference Sequence Database (ViralRefSeq), the Oral Virus Database (OVD), the Pig Virome Database (PVD), and the Gut Virome Database (GVD). To ensure consistency across datasets, only vOTUs and reference database sequences with genome completeness greater than 50% according to the CheckV results were retained for comparison. Pairwise average nucleotide identity (ANI) and alignment fraction (AF) between vOTUs and reference sequences from each database were calculated using Skani v0.2.2 (Shaw and Yu, 2023), which applies an approximate mapping strategy without base-level alignment. Based on Skani output, intersections between vOTUs and each reference database were identified using a custom R script.

### 2.11 Phylogenetic analysis

Phylogenetic analysis of vOTUs was conducted based on the conserved terminase large subunit (*TerL*) gene. *TerL* proteins were identified from vOTUs using HMMER v3.3.2 (Johnson et al., 2010) with hmmsearch against three *TerL* hidden Markov models (PF03237, PF04466, and PF05876) using an E-value threshold of 1 × 10^−5^. A total of 347 open reading frames (ORFs) encoding *TerL* were identified from 18,412 vOTUs. After excluding vOTUs not classified as members of *Caudoviricetes*, 323 *TerL* sequences were retained for phylogenetic analysis. The retained *TerL* protein sequences, together with reference *TerL* sequences, were aligned using MUSCLE v5.2 (Edgar, 2004). Gaps were trimmed using trimAl v1.4 (Capella-Gutiérrez et al., 2009). Maximum-likelihood phylogenetic trees were constructed using IQ-TREE2 v2.3.6 (Minh et al., 2020), with node support assessed using 1,000 ultrafast bootstrap replicates. Two phylogenetic trees were generated for distinct analytical purposes. One tree, focusing on DNA packaging strategy classification, was inferred using the BLOSUM62 + F + R9 model. The second tree, designed to examine the phylogenetic distribution of *TerL* domain architectures, was constructed using the BLOSUM62 + F + R10 model. Additionally, a proteomic phylogenetic analysis was performed to examine the evolutionary relationships among the identified vOTUs. For sequences with > 50% completeness, a proteomic tree was constructed using ViPTreeGen v1.1.2 (Nishimura et al., 2017) and subsequently visualized using the Interactive Tree Of Life (iToL) v7.2 (Letunic and Bork, 2024).

### 2.12 Statistical analysis and visualization

All statistical analyses and visualizations were performed using R v4.4.2. Alpha and beta diversity were calculated using the vegan package based on the relative abundance of vOTUs. For alpha diversity indices (Shannon index and observed richness), comparisons between groups were performed using two-sided Wilcoxon rank-sum tests. For comparisons between domestic pigs and wild boars, *α*-diversity comparisons between host groups were performed using only BAL samples processed with the same membrane pore size (Normal, 0.22 μm). For multiple group comparisons, *p*-values were adjusted using the Benjamini–Hochberg false discovery rate (FDR) method. Beta diversity was assessed using Bray–Curtis dissimilarity and tested with permutational multivariate analysis of variance (PERMANOVA) implemented in adonis2 (vegan) with 999 permutations. Principal coordinate analysis (PCoA) was used to visualize beta diversity patterns. Cumulative species accumulation curves were generated using the specaccum function in vegan. Differential abundance analyses at both the vOTU and gene levels were conducted using two-sided Wilcoxon rank-sum tests applied to RPKM-normalized abundances. Log2 fold change was calculated from group mean values. Statistical significance was defined as *p* < 0.05 combined with |log2 fold change| > 1. Group-specific biomarkers were identified using linear discriminant analysis effect size (LEfSe) with a Kruskal-Wallis cutoff of 0.05 and an LDA threshold of 2.

Visualization was performed using ggplot2, ggsankeyfier, and itol.toolkit packages. Bar plots, boxplots, and volcano plots were generated with ggplot2. Heatmaps were produced using heatmap and pheatmap packages. Venn diagrams and UpSet plots were generated using VennDiagram and UpSetR.

## 3 Results

### 3.1 Characterization of vOTUs in the porcine lungs

In this study, virus-like particle (VLP) enrichment was performed on 49 samples (including 41 BAL, 4 LTE, and 4 LTH) collected from 17 domestic pigs and 20 wild boars under natural conditions. DNA extracted from enriched VLP was sequenced and used for virome analysis. In total, 1,194.6 Gb of raw sequencing data were generated, with an average of 24.38 Gb per sample. After quality control and host DNA removal, approximately 10.0% (~ 119.2 Gb) of the original sequencing data were retained, yielding an average of 2.43 Gb of clean reads per sample for next analyses. The assembled contigs produced an average total length of approximately 61.25 Mbp per sample (Supplementary Figure S1B; Supplementary Table S1). Negative controls produced fewer sequencing reads than experimental samples. Host DNA removal significantly reduced the number of sequencing reads in experimental samples; consistent with previous observation of extensive host contamination in porcine lung microbiome study (Li et al., 2023). In contrast, no significant difference was detected between the raw and clean datasets of negative controls (Supplementary Figure S1C). In addition, 1,297 viral contigs were detected in negative controls, among contigs with ≥ 50% genome completeness, 22 could be taxonomically annotated, including 19 bacteriophages and several *ss*DNA viruses.

Using sequencing data after host DNA removal, we assembled 47,854 viral contigs, most of which were longer than 1.5 kb. After clustering, 37,890 vOTUs were obtained and evaluated using CheckV. In this quality assessment, 3,565 vOTUs met at least 50% completeness, 1,248 were classified as complete, 758 as high-quality, and 1,559 as medium-quality genomes (Supplementary Figure S1D; Supplementary Table S2). These values represent the initial vOTU dataset before contaminant removal, abundance- and prevalence-based filtering, and outlier sample exclusion.

Potential contaminant vOTUs were identified using the “decontam” R package to evaluate its distribution and potential impact among the experimental samples (Supplementary Figure S2A). The total abundance of contaminant vOTUs was significantly higher in PBS controls than in the experimental samples (*p* = 0.001), whereas non-contaminant vOTUs showed significantly higher abundance in experimental samples (*p =* 6.83 × 10^−6^), indicating effective discrimination between background contamination and true viral signals. The prevalence of these contaminant vOTUs remained low, with most detected in fewer than 20% of experimental samples (Supplementary Figure S2B), indicating a limited impact on the downstream analyses. To further minimize potential contamination, vOTUs showing no statistically significant difference in RPKM abundance between the negative controls and experimental samples were removed. The vOTUs exhibited substantial variation in GC content (17.92–73.03%) and genome length (1,301 bp - 663,218 bp). Four vOTUs had large genomes (>100,000 bp) and high GC content (>60%), including one classified as *Adintoviridae* (recently renamed *Eupolintoviridae*), a *ds*DNA viral family reported to infect both invertebrate and vertebrate eukaryotic hosts (Supplementary Figure S2C; Supplementary Tables S2, S3).

Across the dataset, a total of 906 vOTUs were detected in more than 50% of the samples, with RPKM values ranging from 4.136 to 5,348.376. In contrast, 4,345 vOTUs were detected in more than five samples, with RPKM values ranging from 0.291 to 8,343.008. We applied an abundance and prevalence-based filtering strategy. vOTUs detected in at least two samples were retained if their mean RPKM exceeded 0.291, whereas those detected in only one sample were required to exceed 0.582 RPKM (twice the filtering threshold). To further ensure data robustness, two samples (WB7562W04 and WB7562W05) contained extremely few vOTUs (less than 0.1% of total vOTUs) which showed inflated relative abundance estimates (Supplementary Figure S3A) and were removed. Non-contaminant vOTUs were retained for downstream analysis, whereas contaminant vOTUs identified by “decontam” were further filtered based on statistical significance, retaining only those with *p* < 0.05. After removing outliers and combining replicate samples, 35 samples and 18,412 vOTUs were retained as the final dataset for downstream analyses.

**Figure 1:**
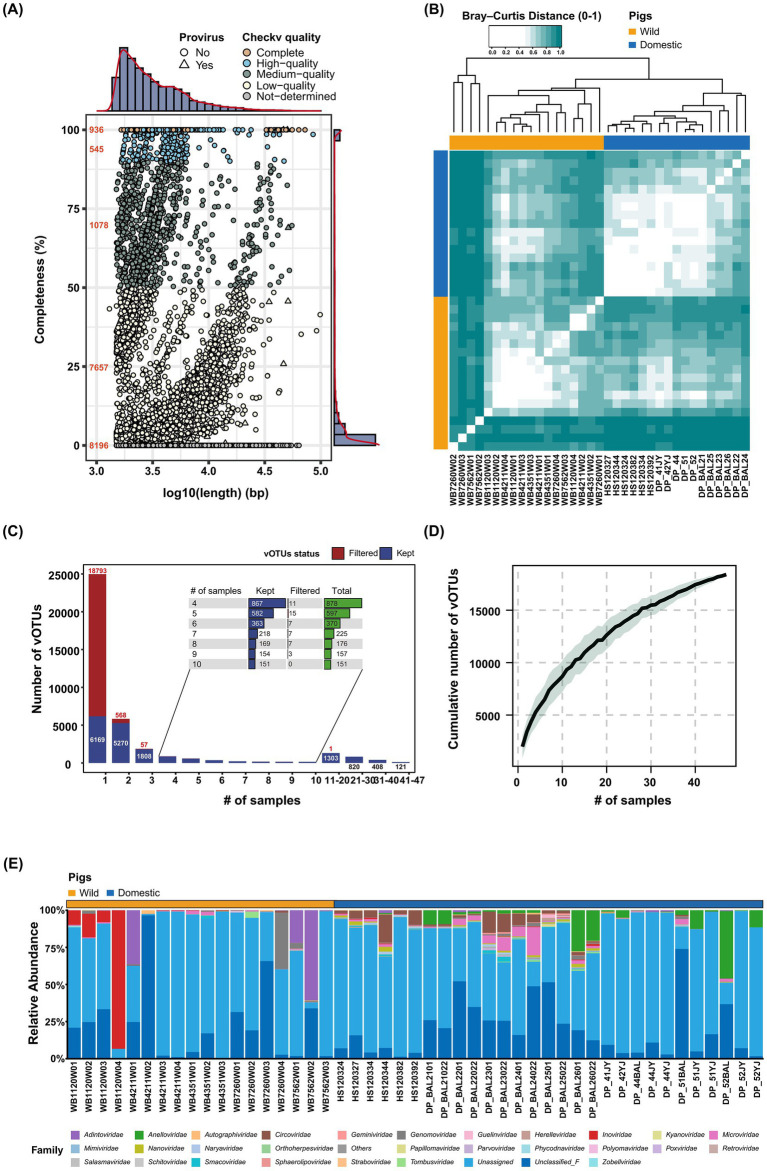
Characteristics of the DNA virome in porcine lungs. **(A)** CheckV completeness versus genome length distribution for all 18,412 vOTUs retained after the full filtering pipeline. Colors indicate CheckV quality categories, and point shape denotes provirus status. Marginal histograms show data distribution of completeness and genome length. Red numeric labels indicate the number of vOTUs with each completeness category. **(B)** Hierarchical clustering of the Bray–Curtis dissimilarity matrix calculated from vOTU profiles separates wild boars and domestic pigs after merging samples. The heatmap shows pairwise Bray-Curtis distances, and the dendrogram represents the hierarchical relationships among samples. Orange and blue bars indicate wild boars and domestic pigs, respectively. **(C)** Distribution of all vOTUs across samples, showing the number of vOTUs detected in different numbers of samples. Red bars indicate filtered vOTUs and blue bars indicate retained vOTUs. Numeric labels indicate vOTU counts. **(D)** Accumulation curve of filtered vOTUs (*n* = 18,412) across samples. The shaded area represents the 95% confidence interval. **(E)** Relative abundance of the top 30 viral families across samples (*n* = 18,412). Stacked bar plots show viral composition in wild boars (orange) and domestic pigs (blue), with colors indicating viral family.

The CheckV quality distributions of vOTUs before and after filtering are shown in Figure 1A and Supplementary Figure S3B, respectively. Heatmaps generated before (Supplementary Figure S3C) and after merging samples (Figure 1B) displayed comparable viral patterns between wild boars and domestic pigs. Notably, although most filtered vOTUs occurred in only one sample, vOTUs with high prevalence were predominantly retained (Figure 1C). The results further showed distinct clustering by host type, reflecting group-specific differences in viral composition. The vOTU accumulation curve increased without reaching a plateau (Figure 1D), indicating that additional sampling would likely reveal further viral diversity and that the current dataset represents only a partial characterization of this community.

### 3.2 Taxonomic classification of the porcine lung DNA virome

Taxonomic classification was conducted for the filtered vOTUs (*n* = 18,412) from porcine lungs samples. In total, 10,819 vOTUs (~ 58.76%) could be assigned to known viral taxa, corresponding to 29 viral orders and 65 viral families, and accumulation curves at both taxonomic levels approached saturation (Supplementary Figures S4A,B). The viral families with the largest numbers of vOTUs included *Microviridae* (*n* = 1,267), *Circoviridae* (*n* = 359), *Smacoviridae* (*n* = 218), *Adintoviridae* (*n* = 216), and *Autographiviridae* (*n* = 195) (Supplementary Figures S4C–F; Supplementary Table S3A). Among these predominant viral families, *Autographiviridae* and *Adintoviridae* are double-stranded DNA (*ds*DNA) viruses, whereas the remaining families are single-stranded DNA (*ss*DNA) viruses. Approximately half of the identified families belonged to the class *Caudoviricetes*. Within this class *Caudoviricetes*, most vOTUs were annotated as order *Unclassified_O* (*n* = 7,793) or family *Unclassified_F* (*n* = 7,355), indicating the substantial unexplored diversity in the porcine lung virome.

The 30 most abundant viral families across all samples were further analyzed based on the vOTU abundance, revealing the compositional differences between wild boars and domestic pigs. Several of these families belonged to the class *Caudoviricetes*, including both unclassified groups and classified groups such as *Straboviridae*, *Schitoviridae*, *Salasmaviridae*, *Kyanoviridae*, *Herelleviridae*, *Guelinviridae*, *Zobellviridae* and *Autographiviridae*. *Microviridae*, a family of small *ss*DNA bacteriophages, was also among the most abundant families across samples. In addition, *Anelloviridae* and *Circoviridae* (both circular ssDNA viruses commonly detected in mammals) were predominantly detected in domestic pigs, whereas *Inoviridae* and *Adintoviridae* were mainly enriched in wild boars (Figure 1E). *Adintoviridae* is a group of *ds*DNA viruses that have been reported to infect both invertebrates and vertebrates.

Among the 18,412 filtered vOTUs, 2,559 had a genome completeness of at least 50% as assessed by CheckV (936 Complete, 546 High-quality, and 1,077 Medium-quality). Of these vOTUs, 1,632 (63.77%) could be assigned to 21 families and 11 classes. The dominant families included *Microviridae* (*n* = 867), *Circoviridae* (*n* = 259), and *Smacoviridae* (*n* = 205), consistent with the taxonomic composition of the full vOTU dataset (Supplementary Table S3). To further characterize these vOTUs, a phylogenetic tree was constructed annotated with viral families, lifestyle categories, CheckV quality, GC content, and genome length (Supplementary Figure S5). This analysis also revealed broad phylogenetic distribution and substantial genomic diversity across the completeness quality.

### 3.3 Impact of membrane pore size and sampling method on the porcine lung DNA virome

**Figure 2:**
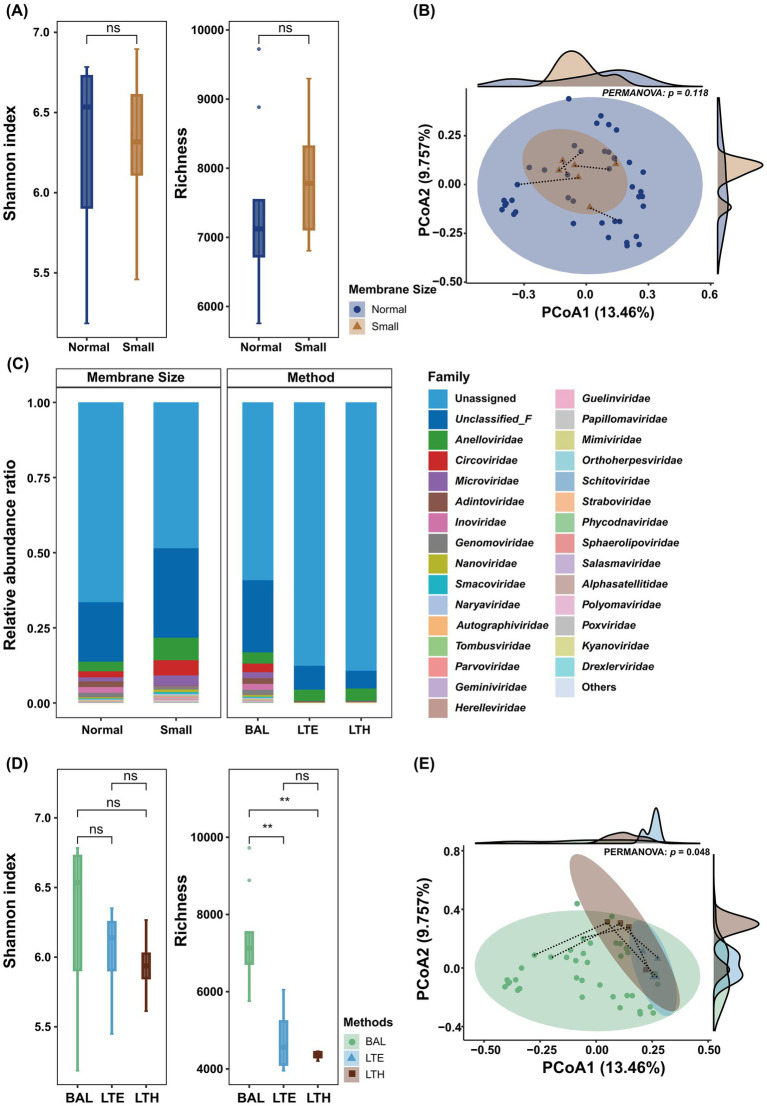
Viral community diversity and composition across different filter membrane pore sizes and sampling methods. **(A,B)** *α*-Diversity and *β*-diversity of the lung virome in bronchoalveolar lavage fluid (BAL) samples filtered using 0.22 μm (normal) and 0.1 μm (small) pore size membranes. **(A)** Shannon index and richness. **(B)** Principal coordinates analysis (PCoA) based on the Bray–Curtis dissimilarity. **(C)** Relative abundance of viral families in samples under different filter pore sizes and sampling methods. **(D,E)** α-Diversity and β-diversity of the lung DNA virome across different sampling methods. **(D)** Shannon index and richness among BAL, LTE, and LTH samples. **(E)** PCoA based on the Bray–Curtis dissimilarity showing composition differences among sampling methods. Statistical significance is indicated as follows: ns, not significant; *p < 0.05; **p < 0.01; ***p < 0.001; ****p < 0.0001.

The impact of filter membrane pore size on viral community composition was evaluated in the VLP enrichment stage, where we used membrane pore sizes of 0.10 μm (Small) and 0.22 μm (Normal) for the final VLPs filtration. All samples included in this pore size comparison were collected using the same sampling method (bronchoalveolar lavage fluid, BAL), with all other experimental parameters and reagents kept the same. No significant differences in *α*-diversity were observed between the two filter groups, including the Shannon index (*p* = 0.52) and observed richness (*p* = 0.38). For *β*-diversity, viral community composition did not cluster according to filter membrane pore size (Figures 2A,B; Supplementary Table S6). The family-level taxonomic composition of vOTUs generated using the two filter sizes showed minimal differences (Figure 2C). Although certain *ss*DNA viruses appeared relatively enriched in the 0.10 μm filter group, Shannon index and richness did not differ significantly between pore sizes, indicating minor effects of pore size on overall diversity. Compared with 0.10 μm filters, 0.22 μm filters allowed smoother liquid passage during sample processing, and it was selected for VLP enrichment of BAL samples.

Viral community composition was also compared across different sample processing methods, including BAL, LTE, and LTH, with all other experimental conditions kept the same. No significant differences were observed in the Shannon index among the three sample processing methods, while observed richness differed significantly. Specifically, BAL samples exhibited significantly higher species richness than LTE or LTH samples (*p*_adj_ = 0.0084, Figure 2D), whereas no significant difference was observed between LTE and LTH (*p*_adj_ = 1.000). These results showed that BAL samples contained a broader range of vOTUs, while overall community evenness remained comparable to tissue-based methods, resulting in similar overall diversity indices. β-diversity analysis using principal coordinate analysis (PCoA) based on Bray-Curtis distances showed slight shifts among groups but no clear separation, indicating broadly similar viral community compositions across sampling methods (Figure 2E; Supplementary Table S4). Further comparison at the viral family level showed that sample processing methods have a greater impact on lung DNA virome composition. Samples processed by LTE and LTH showed lower overall viral abundance than BAL samples, although family level compositions were similar. For example, BAL samples showed higher absolute abundances of *Circoviridae* and several unassigned viral taxa. Overall, these results indicate that the BAL sampling method is more effective for enriching and recovering a greater number of vOTUs than LTE or LTH (Figure 2C).

### 3.4 Virus-host linkages in the porcine lung DNA virome

**Figure 3:**
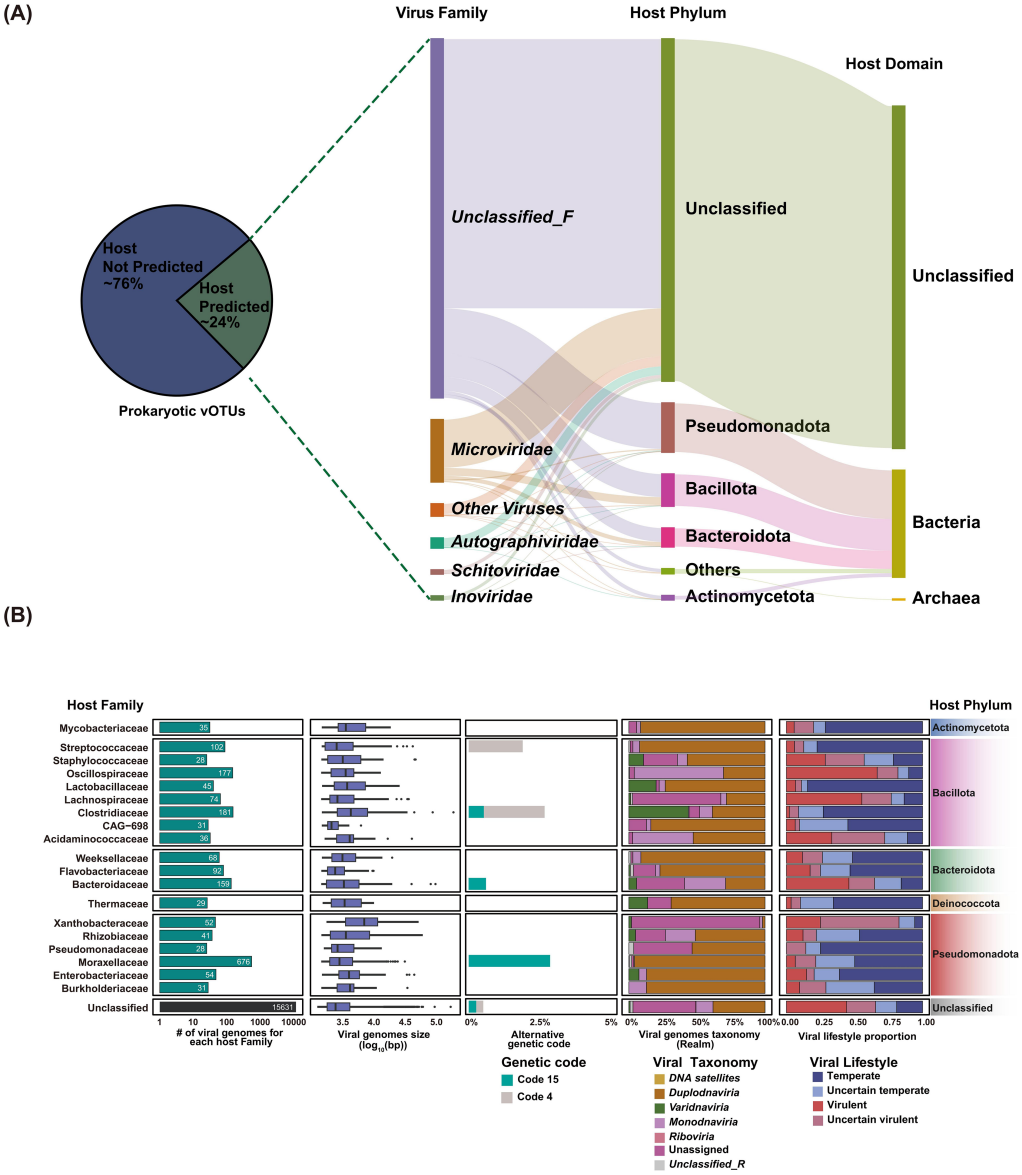
Host prediction and associated characteristics of vOTUs (*n* = 9,202). **(A)** Proportion of vOTUs predicted to infect prokaryotic hosts with or without specific host taxon assignments. The Sankey diagram shows the associations between viral families, predicted host phyla, and host domains. Numbers in parentheses indicated vOTU counts assigned to each taxonomic group. **(B)** Characteristics of vOTUs (*n* = 9,202) predicted to infect prokaryotic hosts stratified by predicted host families, including viral genome size, domain classification, the proportion of viruses predicted by geNomad to use non-standard genetic codes, and viral lifestyle categories. Viral lifestyle categories are defined by the DeePhage score: temperate (score ≤ 0.3), uncertain temperate (0.3 < score ≤ 0.5), uncertain virulent (0.5 < score ≤ 0.7), and virulent (score > 0.7).

Host prediction was performed to characterize virus-host linkages in the porcine lung DNA virome. In our study, iPHoP was used to predict hosts for the 18,412 identified vOTUs. Among these vOTUs, 9,202 showed signals consistent with infection of prokaryotic hosts, and 2,189 (~24%) were confidently assigned to specific host taxa (Supplementary Tables S5–S7; Figure 3A), the remaining vOTUs (~76%) without specific host taxon assignments were still classified at least to the class level. The predicted hosts were primarily assigned to bacterial phyla, commonly detected in the porcine lower respiratory tract, including Pseudomonadota (*n* = 997), Bacillota (*n* = 649), Bacteroidota (*n* = 375), and Actinomycetota (*n* = 77). Among all vOTUs with any host prediction at the domain, most were bacteria (2,183 out of 2,189), whereas archaeal hosts accounted for only 0.27%. Only 524 vOTUs had both confident viral family-level classification and corresponding host prediction (Supplementary Table S7B), representing the subset with the most comprehensive annotations in our dataset and reflecting the limited confident virus-host associations. At the host family level, the most frequently predicted hosts included Moraxellaceae (*n* = 651), Clostridiaceae (*n* = 70), Oscillospiraceae (*n* = 170), Bacteroidaceae (*n* = 93), and Streptococcaceae (*n* = 98). At the genus level, the most commonly predicted hosts included *Acinetobacter* (*n* = 574), *Streptococcus* (*n* = 95), *Prevotella* (*n* = 45), and *Clostridium* (*n* = 37) (Supplementary Table S7B). These genera have been reported to be associated with porcine pulmonary infections, and *Acinetobacter* has been detected in the lower respiratory tract of pigs in a previous microbiome study (Zhou et al., 2023).

### 3.5 Prediction of viral lifestyle in the porcine lung DNA virome

Using DeePhage, the 18,412 vOTUs were classified into four lifestyle categories: virulent (7,457; 40.5%), temperate (4,159; 22.6%), uncertain virulent (3,792; 20.6%), and uncertain temperate (3,004; 16.3%) (Supplementary Figure S6A; Supplementary Table S8). Further visualization of the top 20 predicted host families summarized viral counts, genome sizes, taxonomic classifications, lifestyle categories, and genetic codes, grouped by host phylum (Figure 3B; Supplementary Figures S6B,C). Notably, vOTUs with unclassified hosts vastly outnumbered those assigned to any single family, indicating that a substantial proportion of the virome remains unresolved host-virus linkages. Among classified host families, Moraxellaceae, Bacteroidaceae, Oscillospiraceae, and Clostridiaceae were the most frequently predicted.

Viral genome sizes varied markedly across host families, with the median genome sizes ranging from approximately 1.3 to 195 kb and its IQR spanning approximately 1.9 to 4.5 kb. These differences suggest that variation in viral genomes may be shaped by host family specific ecological characteristics.

In addition to variation in genome size, host-associated differences in viral genetic code usage were also identified. Among viruses predicted to infect Moraxellaceae and Bacteroidaceae, 2.61 and 1.07%, respectively, were inferred to employ genetic code 15 instead of the standard bacterial genetic code 11, and viruses associated with Moraxellaceae were overwhelmingly classified within *Duplodnaviria*. Similarly, among viruses predicted to infect Streptococcaceae (phylum Bacillota), 2.04% were inferred to use genetic code 4. This pattern is consistent with previous reports of stop-codon reassignment in bacteriophages, suggesting that certain viruses infecting Bacillota may utilize alternative genetic codes as an adaptation to host-specific translational systems (Borges et al., 2022).

Regarding viral taxonomy, *Duplodnaviria* was the predominant viral realm across most host families. However, members of *Monodnaviria* were also observed in host families such as Oscillospiraceae and Acidaminococcaceae, highlighting the ecological and phylogenetic diversity of the lung virome. Lifestyle analysis further revealed that virulent viruses predominated in hosts such as Acidaminococcaceae, Lachnospiraceae, and Oscillospiraceae, while temperate viruses were more frequently associated with hosts including Mycobacteriaceae, Lactobacillaceae, Clostridiaceae, and Aeromonadaceae. Collectively, these results indicate the presence of distinct virus-host interaction strategies and underscore the influence of host ecological characteristics on the virome community structure and potential functional roles (Figure 3B; Supplementary Table S7B).

### 3.6 Auxiliary metabolic genes (AMGs) in porcine lung DNA virome

**Figure 4:**
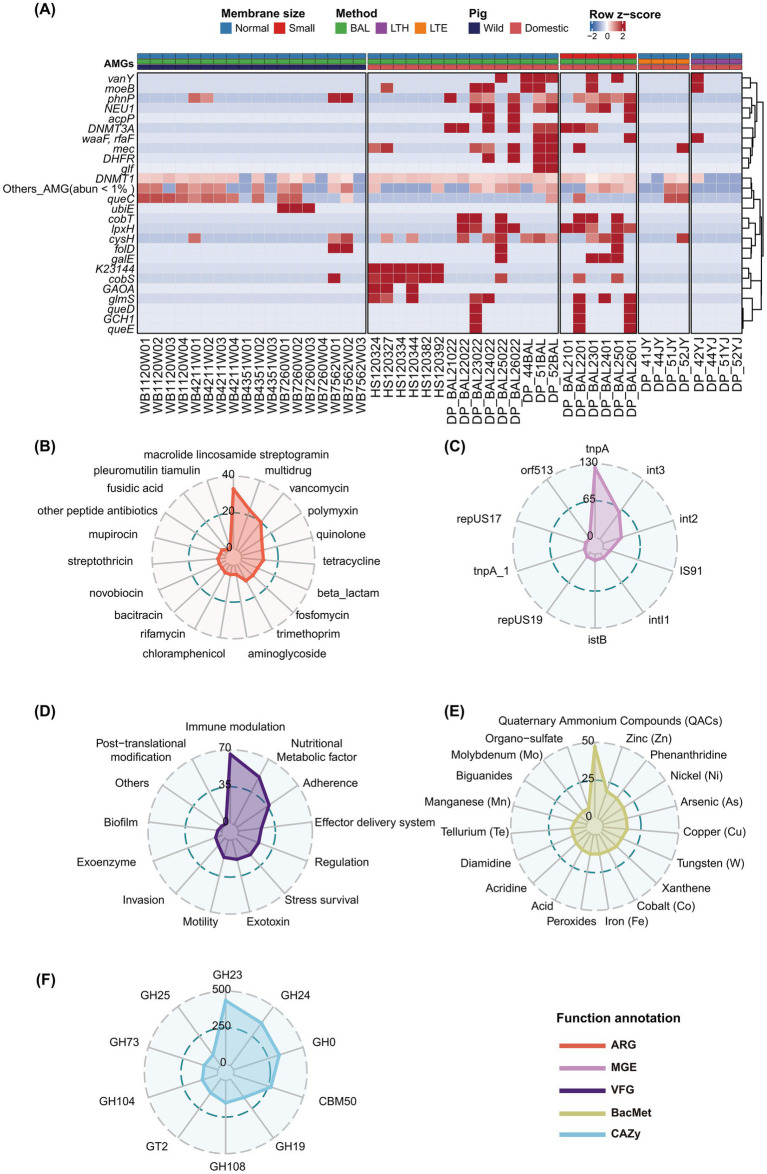
Functional annotation of the porcine lung DNA virome. **(A)** Distribution patterns of auxiliary metabolic genes (AMGs) across samples, grouped by membrane pore size, sampling method, and host type. **(B–F)** Radar plots summarizing major functional gene categories annotated using multiple reference databases. **(B)** Antibiotic resistance genes (ARGs) annotated against the SARG database. **(C)** Mobile genetic elements (MGEs) against the MGEs database. **(D)** Virus-derived virulence factor genes (VFGs) annotated against the VFDB database. **(E)** Metal resistance genes annotated against the BacMet database. **(F)** Carbohydrate-active enzymes annotated against the CAZy database.

Viruses commonly carry auxiliary metabolic genes (AMGs), which play important roles in microbially mediated biochemical processes and have been implicated in a range of host associated biological functions. A total of 191 vOTUs carrying putative AMGs were identified, comprising 40 AMG types spanning 31 metabolic pathways across 11 major metabolism categories. These AMGs were most highly enriched in pathways related to cysteine and methionine metabolism (*n* = 80), folate biosynthesis (*n* = 18), sulfur metabolism (*n* = 14), and the one-carbon pool by folate (*n* = 13). Additional pathways included glycan biosynthesis, lipid and energy metabolism, biosynthesis of other secondary metabolites, and carbohydrate metabolism (Supplementary Tables S9, S10). Overall, this distribution indicates that viral AMGs are involved in a broad range of host associated metabolic processes. These AMGs included *cysH*, *queC*, *queD*, *queE*, *cobS*, *moeB*, *glmS*, *DNMT1*, *DNMT3A*, *DHFR*, and *mec*. Genes associated with carbohydrate metabolism (e.g., *glmS*, encoding glutamine-fructose-6-phosphate transaminase) and sulfur and phosphorus cycling (e.g., *cysH*, encoding phosphoadenosine phosphate sulfate reductase, and *moeB*, encoding molybdenum cofactor biosynthesis) were frequently detected in samples (Figure 4A). In addition, a multivariate analysis based on Bray-Curtis distances indicated variation in AMG composition among samples (PERMANOVA, R^2^ = 0.34, *p* = 0.001). Specifically, *cysH* encodes 3′-phosphoadenosine-5′-phosphosulfate reductase, which catalyzes the reduction of activated sulfate to sulfite, a key step in assimilatory sulfate reduction. In addition, *cobS* and *queE* are involved in cofactors and vitamins biosynthesis, whereas *queC*, *queD*, and *queE* are key components of the folate (vitamin B9) biosynthetic pathway. Notably, *DNMT*-like genes were identified on a subset of vOTUs and were annotated as DNA methyltransferases; however, their origin and functional roles remain uncertain and require cautious interpretation. In this study, several *DNMT*-like genes were located on viral contigs together with predicted viral genes such as capsid proteins and terminase subunits, but their functional roles in the porcine lung virome remain unclear.

### 3.7 Functional annotations of the porcine lung DNA virome

A total of 105,826 hypothetical proteins were predicted from vOTUs and annotated against five functional databases, including SARG, MGEs, VFDB, BacMet, and CAZy. Counts (*n*) represent the number of annotation hits within each functional category. Based on SARG annotations, 902 antibiotic resistance genes (ARGs) were identified. The most prevalent resistance categories included macrolide-lincosamide-streptogramin resistance (*n* = 33), multidrug resistance (*n* = 23), and vancomycin resistance (*n* = 20) (Figure 4B; Supplementary Table S11). Annotation against the MGEs database identified 27 types of mobile genetic elements (MGEs). Among these, the transposase gene *tnpA* (*n* = 123) and the integrase gene *int3* (*n* = 58) were the most abundant (Figure 4C; Supplementary Table S11). These elements have been reported to play important roles in resistance gene transposition. In addition, previous studies have shown that a substantial proportion of ARGs detected in the pig metagenome are not transcriptionally active (Wang et al., 2020). Accordingly, although multiple ARGs were detected in the porcine lung virome and are interpreted as putative annotations associated with viral contigs, their contribution to antimicrobial resistance in the respiratory environment remains unclear.

Virulence factor genes (VFGs) were also detected in the porcine lung virome. These genes were primarily associated with immune modulation (*n* = 66), nutritional / metabolic factors (*n* = 51), adhesion (*n* = 37), and effector delivery systems (*n* = 22), consistent with previous reports on viral virulence factors (Figure 4D; Supplementary Table S11). In addition, annotation against the BacMet database identified 143 metal resistance genes, among which the genes conferring resistance to zinc (Zn, *n* = 19) and copper (Cu, *n* = 16) were particularly prominent (Figure 4E; Supplementary Table S11).

Annotation against the CAZy database identified 330 non-redundant carbohydrate-active enzyme genes. The most frequent CAZyme family was GH23 (*n* = 435, annotation hits; glycoside hydrolases), suggesting that a substantial proportion of lung-associated viruses are bacteriophages encoding lysozyme-related enzymes that may contribute to peptidoglycan degradation (Figure 4F). Other CAZyme families included CBM50 (*n* = 270, annotation hits; carbohydrate-binding modules), GT2 (*n* = 119, annotation hits; glycosyltransferases), and CE4 (*n* = 3, annotation hits; carbohydrate esterases), indicating that viruses may participate in carbon cycling through degradation of complex polysaccharides in the porcine lung microenvironment (Supplementary Table S11).

### 3.8 Phylogenetic analysis of the porcine lung virome based on the conserved gene *TerL*

**Figure 5:**
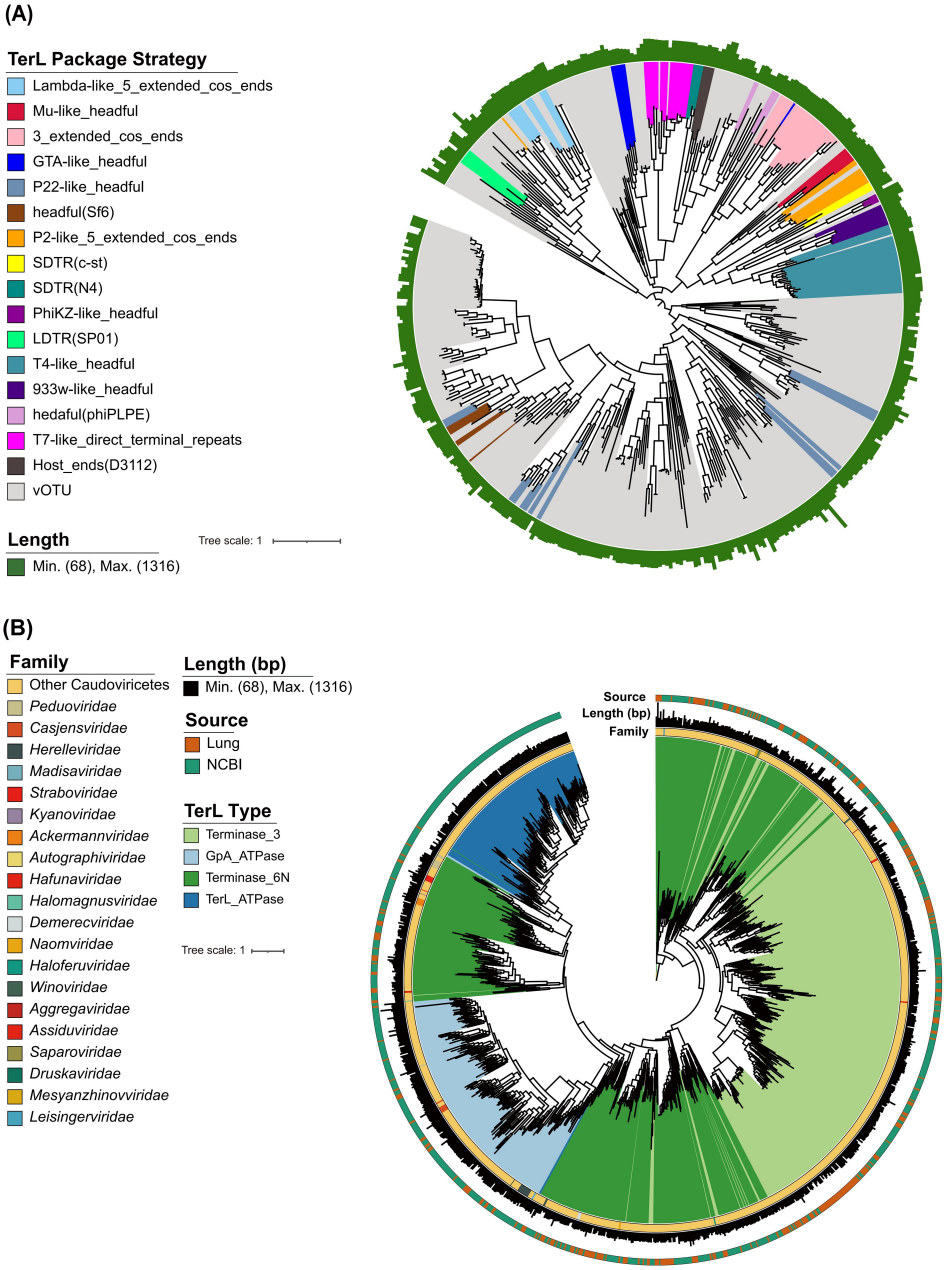
Phylogenetic analysis of *TerL*-carrying vOTUs. **(A)** Maximum-likelihood phylogenetic tree of *Caudoviricetes* constructed using terminase large subunits (*TerL*) sequences. Colors represent different *TerL* DNA packaging strategies, and the outer bar plot represents genome size. **(B)** Maximum-likelihood phylogenetic tree based on *TerL* sequences. Colors indicate different *TerL* domains. Viral family is indicated by the inner circle, and the source *TerL* domain sequences are indicated by the outer circle. The length of *TerL* domain sequences ranges from 68 to 1,316 bp.

Among the 18,412 vOTUs identified in the porcine lung virome, 10,819 can be classified, and the majority (7,855 of 10,819; 72.60%) were assigned to the class *Caudoviricetes* (Supplementary Table S12). *Caudoviricetes*, a class of dsDNA tailed bacteriophages, are frequently detected viruses in other microbiome studies. Because the terminase large subunit (*TerL*) gene is conserved among head-tail phages (Rao and Feiss, 2015), a *TerL*-based phylogenetic analysis was performed to examine the distribution of DNA packaging strategies within *Caudoviricetes* in the porcine lung virome. A total of 347 open reading frames (ORFs) encoding *TerL* proteins were identified from the 18,412 vOTUs. The limited recovery of *TerL* likely reflects the presence of *ss*DNA viruses and the fragmented viral genome assemblies. After excluding vOTUs not classified as *Caudoviricetes*, 323 *TerL*-carrying vOTUs, together with reference *TerL* sequences (Supplementary Table S12), were retained for subsequent analysis. As shown in Figure 5A, *TerL*-carrying vOTUs were distributed across 16 distinct lineages associated with different DNA packaging strategies. While most sequences clustered within previously recognized *Caudoviricetes* groups, several formed distinct branches, reflecting substantial phylogenetic diversity among head-tail phages in the porcine lung virome.

To investigate the phylogenetic distribution of *TerL* domain architectures, a comprehensive phylogenetic tree was constructed using 323 *TerL* sequences from the porcine lung-associated *Caudoviricetes* together with 791 *TerL* reference sequences retrieved from NCBI (Supplementary Table S13). The phylogenetic analysis grouped the porcine lung viral sequences into multiple divergent lineages within *Caudoviricetes* (Figure 5B). Distinct *TerL* domain architectures, including Terminase_3, GpA_ATPase, Terminase_6N, and TerL_ATPase, were distributed across clearly separated phylogenetic branches and lung-derived sequences broadly represented across these domain types. Quantitative phylogenetic analysis based on *TerL* sequences showed that the median root-to-tip branch length of lung-derived *TerL* vOTUs was significantly shorter than that of reference sequences (Figure 5B, Wilcoxon rank-sum test, *p* = 2.22 × 10^−16^), suggesting that these sequences are positioned closer to the basal nodes of the *Caudoviricetes TerL* phylogeny than the available reference sequences. However, this pattern should be interpreted cautiously, as limited reference diversity may affect phylogenetic placement. Consistently, lung-derived vOTUs accounted for 41.1% of the sequences within the root-proximal 5% of the phylogeny and 38.4% within the root-proximal 10%, indicating enrichment near the root. From a topological perspective, a substantial proportion of *TerL*-carrying vOTUs formed long-branched and deeply rooted clades near the base of the tree, indicating divergent phylogenetic positions within the current *TerL* framework. However, these placements should be interpreted cautiously, as limited reference diversity and substantial sequence divergence may contribute to their apparent basal positions. These sequences may represent divergent host-associated viral lineages in the porcine respiratory environment. Altogether, the diversity of *TerL* domains highlights the evolutionary complexity of the *Caudoviricetes* community in the porcine lung.

### 3.9 Comparison of the porcine lung DNA viral communities between domestic pigs and wild boars

**Figure 6:**
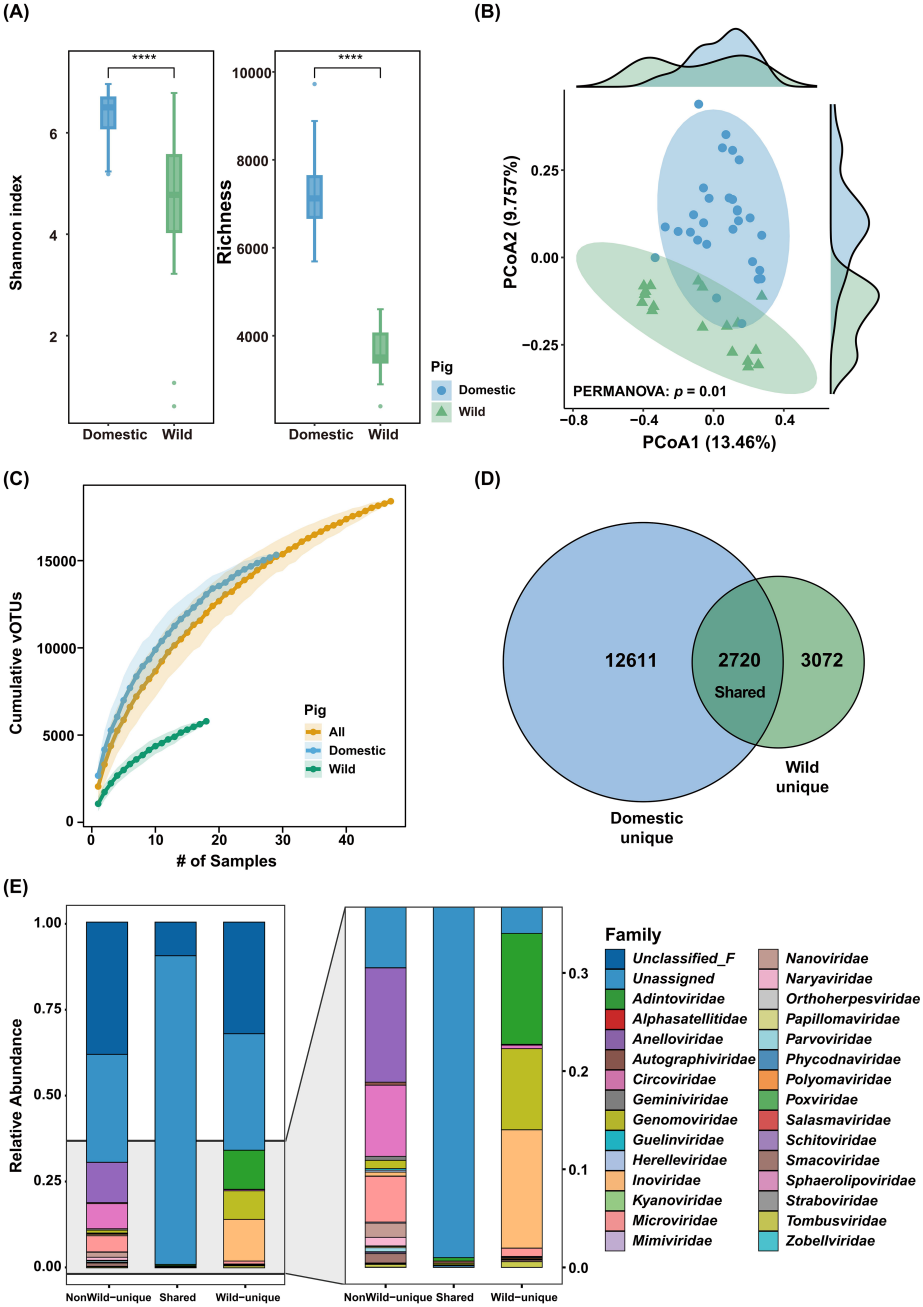
Comparison of lung DNA virome between domestic pigs and wild boars. **(A,B)** α diversity and β-diversity of the lung DNA virome between domestic pigs and wild boars. **(A)** Shannon index and richness of viral communities. **(B)** Principal coordinates analysis (PCoA) based on the Bray–Curtis dissimilarity. *, *p* < 0.05; **p < 0.01; ***p < 0.001; ****p < 0.0001. **(C,D)** Diversity cumulative and distribution patterns of vOTUs in domestic pigs and wild boars. **(C)** Accumulation curves showing the cumulative vOTU counts detected in all pigs, domestic pigs, and wild boars. **(D)** Venn diagram showing the numbers of vOTUs unique in domestic pigs, unique to wild boars, and shared between the two groups. **(E)** Family-level taxonomic composition of group-specific and shared vOTUs.

Lung-associated samples were analyzed to compare lung DNA viromes between wild boars and domestic pigs. *α*-diversity analysis showed significantly higher Shannon index (*p* = 3.33 × 10^−5^) and species richness (*p* = 1.15 × 10^−6^) in domestic pig samples compared with wild boar (Figure 6A), indicating higher viral diversity in domestic pigs. Principal coordinate analysis (PCoA) based on Bray-Curtis distance showed clear separation between the two host groups, reflecting significant differences in viral community composition (Figure 6B).

Accumulation curves showed that the number of observed vOTUs in both domestic pigs and wild boars increased continuously with sample size without reaching a plateau, with domestic pigs consistently exhibiting higher vOTU counts than wild boars (Figure 6C). These results indicate that the lung viral community has not yet been fully captured in either host group, consistent with the higher α-diversity observed in domestic pigs. Venn diagram analysis showed that although 2,720 vOTUs were shared between the two groups, many unique vOTUs were detected in domestic pigs (*n* = 12,611) and wild boars (*n* = 3,072) (Figure 6D), further suggesting distinct virome compositions between host types. This difference remained evident when sample sizes were balanced between groups. Such differences may relate to distinct environmental pressures experienced by domestic pigs and wild boars, including influences of artificial breeding and vaccination in domestic pig populations. Overall, these results reveal observable differences in porcine lung DNA virome diversity and community composition between the domestic pigs and wild boars under the present study design, which may reflect combined effects of host background, geographic variation, and environmental factors.

### 3.10 Comparative analysis of taxonomy and differential abundance in the porcine lung DNA virome between domestic pigs and wild boars

**Figure 7:**
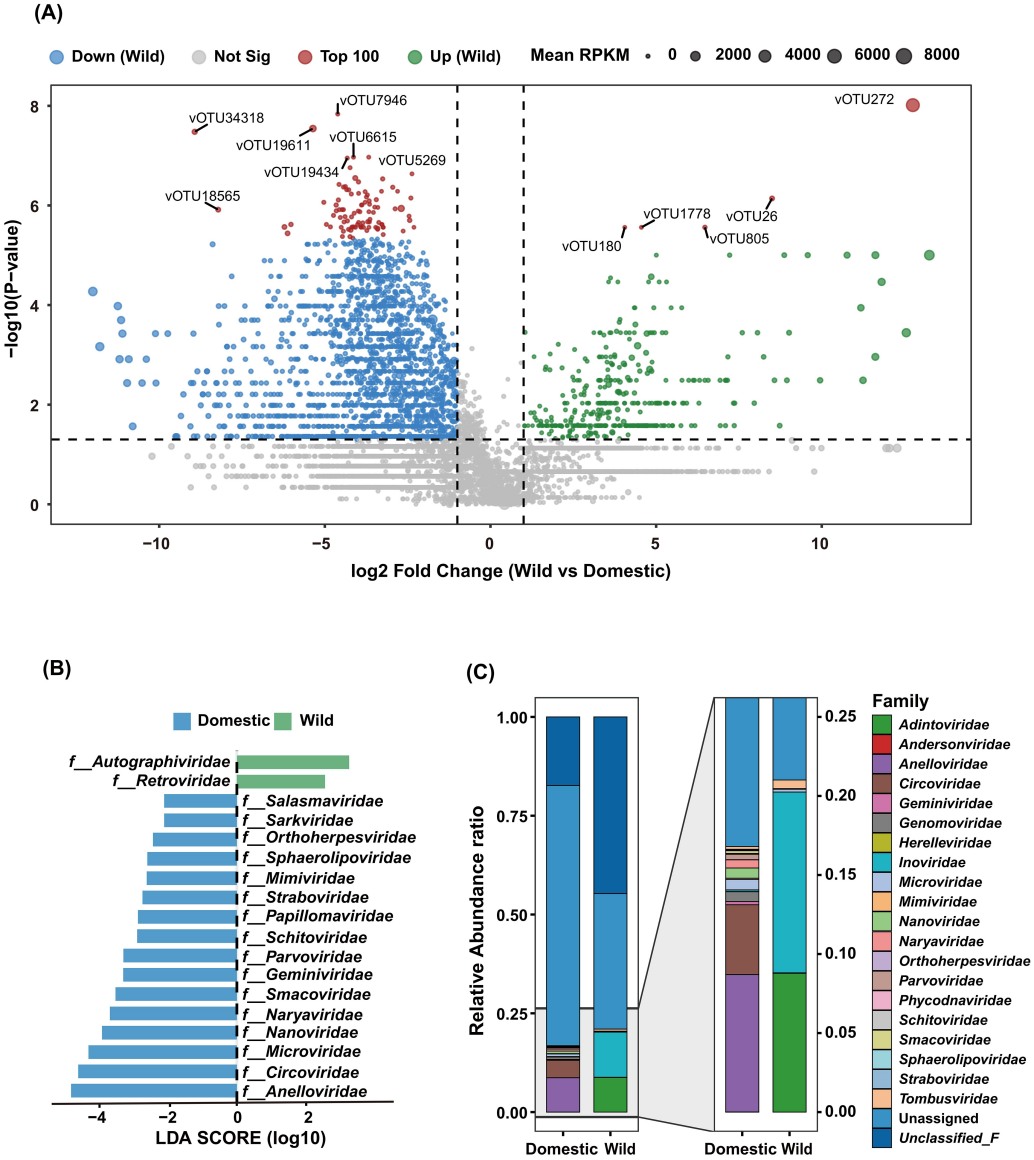
Differential analysis of lung DNA virome between domestic pigs and wild boars. **(A)** Volcano plot showing differentially abundant vOTUs between domestic pigs and wild boars. The *x*-axis represents the log2 fold change (wild versus domestic), and the *y*-axis indicates the −log_10_-transformed *p*-value. Colored points denote vOTU enrichment direction. Selected highly abundant and significant vOTUs are labeled. (B, C) Taxonomic differences at the viral family level between domestic pigs and wild boars. **(B)** Linear discriminant analysis effect size (LEfSe) analysis identifying viral families with significant differential abundance between domestic pigs and wild boars. **(C)** Relative abundance profiles of viral families in domestic pigs and wild boars, highlighting group-specific compositional differences.

To further characterize compositional differences in lung viromes between domestic pigs and wild boars, vOTUs were taxonomically classified and compared at the family level (Figure 6E). The vOTUs shared between the two host groups were predominantly composed of unassigned viruses, *Adintoviridae*, and *Anelloviridae*, suggesting that these viral families represent common components of the lung virome in both hosts. The vOTUs unique to domestic pigs displayed higher diversity at the viral family level, with notable contributions from *Anelloviridae*, *Circoviridae*, and *Microviridae*. In contrast, vOTUs unique to wild boars were characterized by enrichment of *Adintoviridae*, *Genomoviridae*, and *Inoviridae*, indicating distinct viral community signatures associated with different host types. To assess differences in viral taxon abundance between the two host groups, differential abundance analysis was performed using vOTU level read counts. As shown in the volcano plot (Figure 7A), a substantial number of vOTUs were significantly enriched in the wild boar group (green) or the domestic pig group (blue). In total, 372 vOTUs (2.0%) were significantly upregulated in wild boars, whereas 2,294 vOTUs (12.5%) were significantly downregulated. These differences were observed after abundance normalization and filtering. Among the top 100 ranked by effect size and abundance, highly abundant vOTUs were highlighted in red. Detailed information on differentially abundant vOTUs is provided in Supplementary Table S14. In addition, LEfSe analysis identified several viral families significantly associated with each host group (Figure 7B), including *Autographiviridae* and *Retroviridae* enriched in wild boars, whereas *Anelloviridae*, *Circoviridae*, and *Microviridae* served as biomarkers for domestic pigs. Among the top 30 viral families, the relative abundances of *Adintoviridae* (primarily infecting vertebrates) and *Inoviridae* were higher in wild boars, whereas *Circoviridae* and *Anelloviridae* were more abundant in domestic pigs (Figure 7C). Together, these differences reflect pronounced host associated variation in lung DNA virome composition and structure.

### 3.11 Comparative analysis of viral functional genes and lifestyle between wild boars and domestic pigs

**Figure 8:**
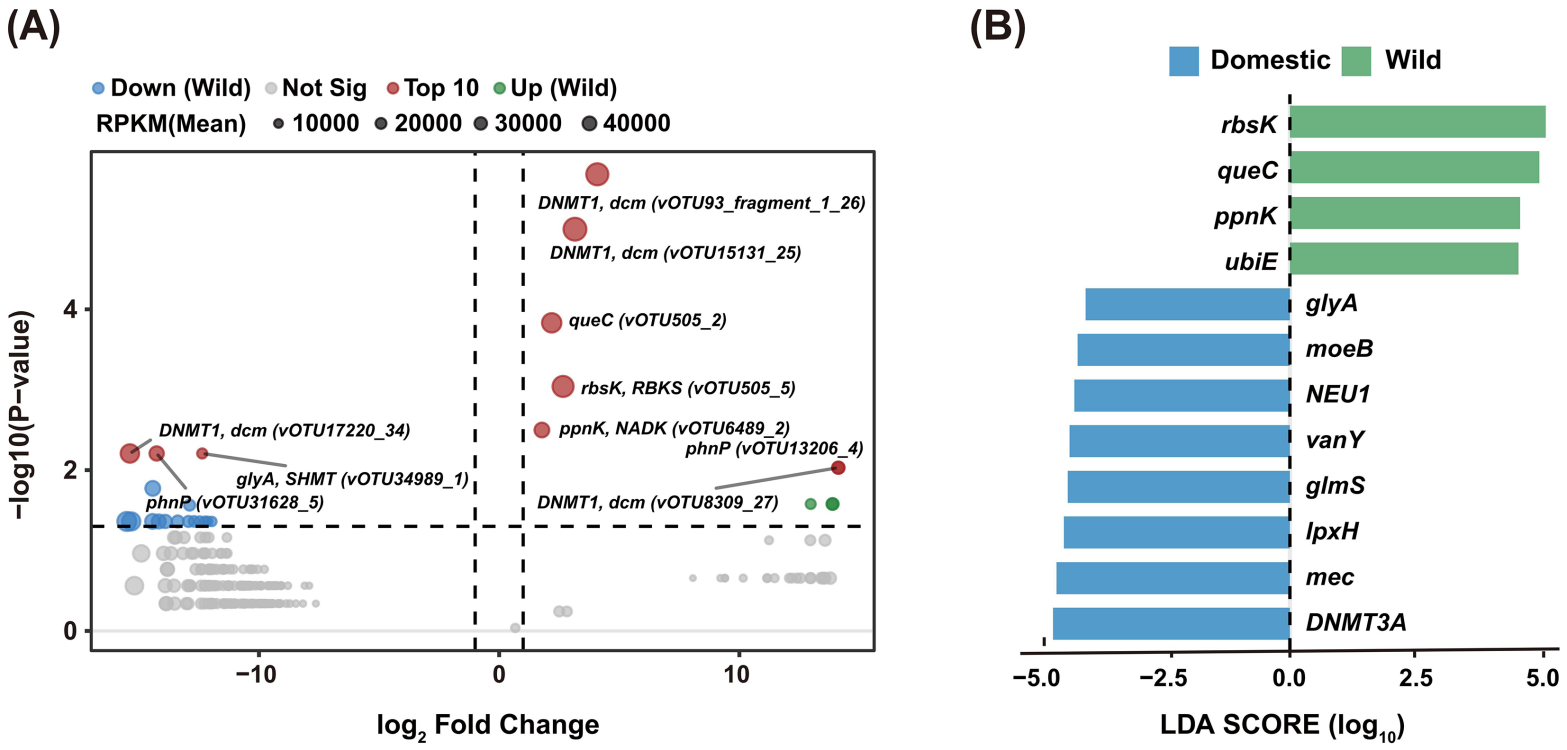
Differential analysis of viral functional genes between domestic pigs and wild boars. **(A)** Volcano plot showing viral functional genes with differential abundance between domestic pigs and wild boars. The *x*-axis represents the log_2_ fold change, and the *y*-axis indicates the −log_10_-transformed *p*-value. Points are colored indicating enrichment direction, and point size reflects mean RPKM. Selected highly abundant and statistically significant genes are labeled. **(B)** Linear discriminant analysis effect size (LEfSe) analysis identifying viral functional genes significantly enriched in domestic pigs or wild boars. Bars indicate LDA scores (log_10_), reflecting the magnitude of differential enrichment between groups.

To further characterize differences in viral functional gene profiles between wild boars and domestic pigs, differential abundance analysis was performed based on annotated viral genes (Figures 8A,B). Several viral functional genes showed significant differences between the two host groups. Genes involved in nucleotide and coenzyme metabolism, including *rbsK*, *queC*, and *ppnK*, showed higher abundance in wild boars, whereas *DNMT1*, *glyA*, and *phnP* were more abundant in domestic pigs. A subset of AMGs, including *DNMT1* and *phnP*, was detected in both host groups and was associated with functions related to nucleotide metabolism, coenzyme synthesis, DNA methylation, and glycosaminoglycan metabolism. Consistent with these results, LEfSe analysis identified functional genes related to nucleotide biosynthesis (*rbsK*, *queC*) and coenzyme metabolism (*ppnK*) as more abundant in wild boars, whereas genes associated with DNA methyltransferases (*DNMT3A*) and glycan metabolism (*lpxH*) were more abundant in domestic pigs (Figure 8B). Notably, *DNMT3A* is considered a host-derived gene, and its enrichment reflects differences observed at the functional annotation level rather than confirmed viral gene content. Regarding viral lifestyle, *α*-diversity analysis showed that domestic pigs had significantly higher Shannon indices than wild boars across all predicted viral lifestyle categories except the uncertain virulent group (Supplementary Figure S7A). This pattern indicates the higher complexity of lung-associated viral communities in domestic pigs. To explore factors contributing to this difference, differential abundance analysis was performed using taxonomic information (Supplementary Figure S7B). Viral lifestyle analysis showed distinct enrichment patterns between the two host groups. Wild boars were mainly enriched in temperate bacteriophages, with family *Inoviridae* as representative taxa, consistent with relatively stable host-virus linkages. In contrast, domestic pig samples were enriched in viral groups dominated by virulent lifestyles, primarily including *Microviridae*, *Circoviridae*, and unclassified viral vOTUs. Previous studies have shown that virulent and temperate phages can display different responses to ecological variation associated with the host environment (Gao et al., 2024). Overall, these results indicate that viral lifestyle differs significantly between wild boars and domestic pigs, with host type associated with differences in lung-associated viral community composition in pigs.

### 3.12 Comparative analysis of porcine lung DNA viral sequences with known viral reference databases

To characterize the novelty of the porcine lung DNA virome, we conducted a comparative analysis between viral sequences obtained from porcine lung samples and five viral reference databases, including MGV, ViralRefSeq, OVD, PVD, and GVD. For consistency across datasets, analyses were restricted to 2,559 vOTUs with genome completeness of at least 50%. Sequences with completeness below 50% were removed from MGV and ViralRefSeq datasets based on CheckV assessments, whereas datasets from OVD, PVD, and GVD were used without further filtering, as they already comprised vOTUs with completeness ≥50%.

**Figure 9:**
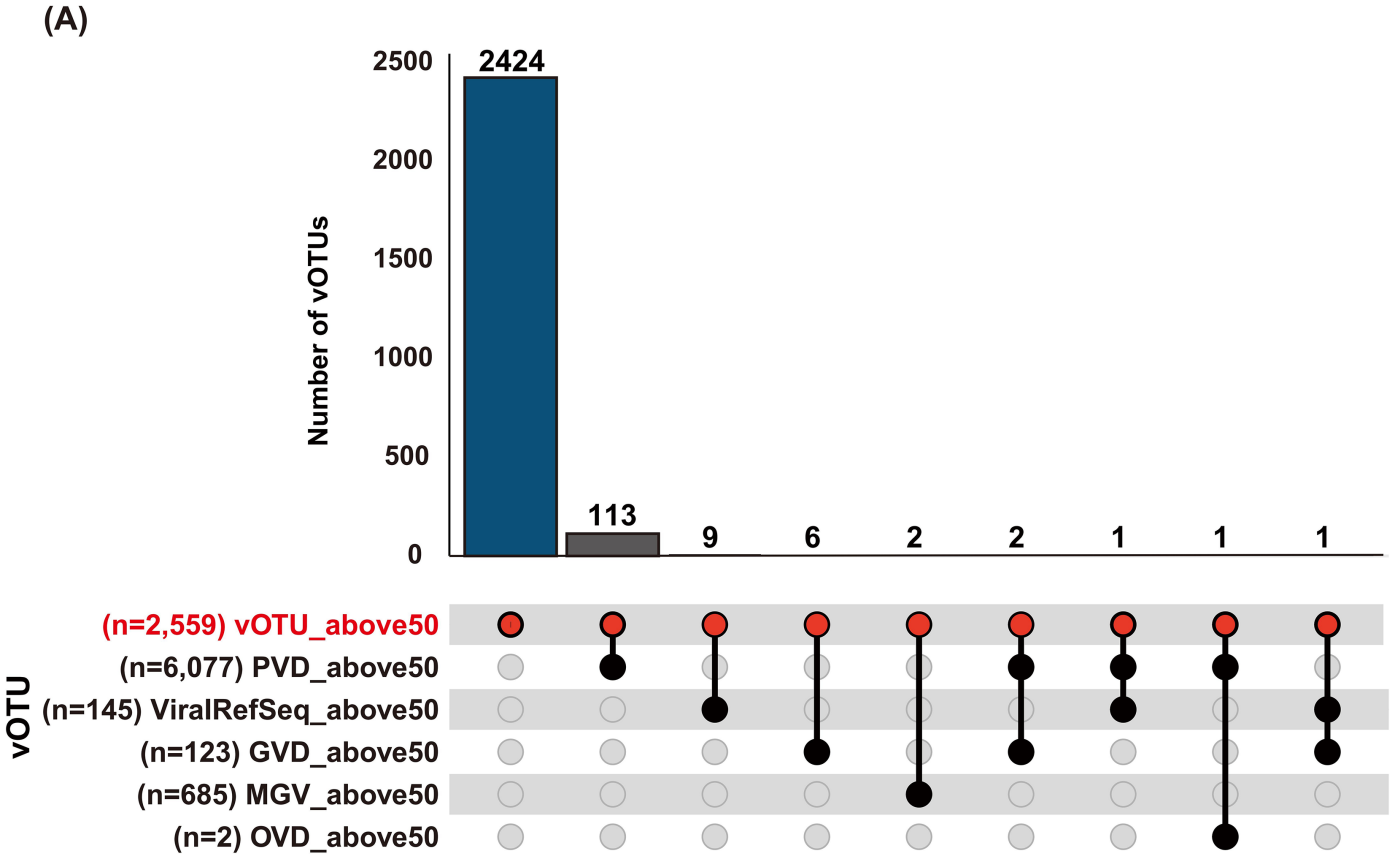
Comparison of porcine lung vOTUs with viral reference databases. UpSet plot showing intersections between the 2,559 vOTUs with genome completeness ≥50% (complete, high-quality, and medium-quality with MIUVIG) and viral reference databases (PVD, ViralRefSeq, GVD, MGV, and OVD). Bars represent the size of each intersection, and connected dots indicate the specific combinations of datasets contributing to each intersection.

Comparative analyses showed that most porcine lung viral sequences differed substantially from viral genomes represented in current databases. Approximately 95% (2,424 out of 2,559) of vOTUs with genome completeness ≥50% did not cluster with any reference viral sequences in the databases at the species level (Figure 9), indicating a large proportion of previously uncharacterized putative viral sequences in porcine lungs. Across all pairwise comparisons with external datasets (PVD, ViralRefSeq, GVD, MGV, and OVD), the numbers of shared ≥50%-completeness vOTUs were consistently low across databases (117, 11, 9, 2, and 1, respectively), representing less than 5% of the total. At the family level, the 117 shared vOTUs were mainly unclassified viruses (*n* = 41), followed by *Circoviridae* (*n* = 25), *Microviridae* (*n* = 18), and *Smacoviridae* (*n* = 17). Together, these results support the presence of putative viral novelty in the porcine lung virome, while also reflecting incomplete reference database coverage.

## 4 Discussion

To investigate lung-associated viral communities in pigs, we collected 49 samples, including 41 bronchoalveolar lavage fluids, 4 lung tissue extrusion fluids, and 4 lung tissue homogenates, from 17 domestic pigs and 20 wild boars. Following virus-like particle (VLP) enrichment and virome sequencing, we identified 18,412 vOTUs after quality control and contamination removal. Approximately 94.7% of these vOTUs lacked species-level matches in viral reference databases, suggesting that the porcine lung DNA virome remains largely uncharacterized and underrepresented in current viral reference databases. This pattern should be interpreted cautiously because current reference databases remain limited for mammalian respiratory viromes. Studies focusing on the porcine lung virome remain scarce. For example, although a nationwide survey identified pathogens such as porcine reproductive and respiratory syndrome virus (PRRSV) and porcine circovirus (PCV) in swine lungs (Burrai et al., 2023), it did not include community-level virome profiling. Accordingly, vOTUs without clear taxonomic assignments were interpreted cautiously as putative viral sequences. Our study presents one of the first catalogs of the porcine lung DNA virome, revealing that *Caudoviricetes* (~72%) were the dominant classified viruses and diverse lineages were present within the *TerL*-based *Caudoviricetes* phylogeny. This finding is consistent with previous reports of high viral diversity in the human lung virome (Liang and Bushman, 2021). However, the phylogenetic placement of long-branched *TerL* sequences should be viewed with caution.

To evaluate the impact of VLP enrichment methods on community composition, we compared two different filter pore sizes (0.10 μm versus 0.22 μm) and three sample processing methods (BAL, LTE, and LTH). No significant differences in α- or *β*- diversity were observed between the two filter sizes, consistent with aquatic and viral metagenome studies showing minimal impact of filter pore size (e.g., 0.22 versus 0.45 μm) on viral community (Hegarty et al., 2022). Among sampling methods, BAL samples exhibited significantly higher species richness than LTE and LTH, suggesting that BAL may better capture viral community diversity in lung microbiome research.

Through host prediction, we found that approximately 24% of vOTUs with genome completeness ≥50% were assigned to putative bacterial hosts, predominantly belonging to *Pseudomonadota*, *Bacillota*, and *Bacteroidota*. This host distribution mirrors with the previous study by Dickson and Huffnagle, who identified *Pseudomonadota*, *Firmicutes* (*Bacillota*), and *Bacteroidota* as dominant phyla in the healthy lung microbiota (Dickson and Huffnagle, 2015), and supports the ecological association between viruses and their bacterial hosts in the porcine lung niche.

We also identified putative viral auxiliary metabolic genes (AMGs) in the porcine lung virome, including genes annotated with sulfate reduction (*cysH*), cofactor and vitamin biosynthesis (*queE*, *cobS*), and DNA methylation (*DNMT1*, *DNMT3A*). Because *DNMT* genes are commonly found in eukaryotic genomes, these annotations should be interpreted cautiously, as metagenomic assemblies cannot completely exclude potential host-derived sequences or contamination. AMGs have been extensively reported in marine viral communities, where they may enhance host metabolism and facilitate viral replication (Hurwitz and U’Ren, 2016). Similarly, AMGs carried by porcine lung viruses may modulate host metabolic and epigenetic pathways, indicating that these viruses play an active ecological role in the lung ecosystem. Together, these results suggest that porcine lung viruses may influence host metabolic and regulatory processes in addition to infection, potentially interacting with metabolic and immune networks of their bacterial hosts, as observed for AMGs in marine systems. These findings provide insights into host-virus-microbiome interactions in swine health and disease.

Additionally, a limited number of ARGs and VFGs were identified within viral sequences. Although the number of ARG annotation hits were limited, macrolide- lincosamide-streptogramin (MLS) and vancomycin resistance genes were detected, some of which were associated with mobile genetic elements (MGEs) such as integrases and transposases. This observation suggests the potential for horizontal transfer of viral ARGs, consistent with previous reports from swine gut and environmental viromes and their ecological relevance in livestock settings (Zhou et al., 2023). In addition, VFGs encoded on viral contigs were associated with functions related to adhesion, immune evasion, and nutrient acquisition, potentially influencing host-microbiota homeostasis and disease susceptibility.

In our study, we compared the composition and function of the lung DNA viromes between wild boars and domestic pigs. We identified substantial divergence in these lung DNA viromes: *Adintoviridae* and *Genomoviridae* were more abundant in wild boars, whereas *Circoviridae* dominated in domestic pigs. Domestic pigs exhibited higher viral diversity and harbored more population-specific vOTUs. However, because geographic location and host-related factors such as age, diet, and environmental exposure were not controlled in this study, these observed differences should be interpreted as descriptive associations within the present sampling framework rather than evidence of host-specific effects. This pattern aligns with findings in pig gut microbiome studies, which reported higher microbial diversity in farmed pigs due to anthropogenic influences (Bae et al., 2025). Notably, AMGs enriched in domestic pigs were associated with regulatory functions such as DNA methylation and glycan metabolism, while those enriched in wild boars linked to core metabolic processes (e.g., nucleotide and coenzyme biosynthesis). The differences between domestic pigs and wild boars may therefore reflect the combined influence of sampling background, geographic variation, and environmental factors.

This study has several limitations that should be considered. First, the number of samples was relatively small, resulting in the lung-associated viral diversity not reaching saturation. Future studies should include more samples to thoroughly characterize the porcine lung virome. Second, although vOTUs were stratified using MIUVIG quality criteria, Medium-quality vOTUs remain partially reconstructed genomes. Therefore, results related to Medium-quality or unassigned vOTUs should be interpreted cautiously, especially for viral novelty, functional annotation, and ecological patterns. Third, functional validation experiments were not performed. Future studies incorporating viral culture approaches and functional assays will be important for elucidating the mechanisms through which viruses influence the porcine lung microbiota. Fourth, Multiple Displacement Amplification (MDA) may introduce amplification bias, particularly favoring circular DNA viruses and potentially affect relative abundance estimates and comparisons. In addition, vMAG reconstruction using vRhyme (Kieft et al., 2022) with three coverage strategies did not yield high-quality bins. This may be related to the predominance of small DNA viruses, such as *Microviridae*, *Circoviridae*, *Smacoviridae*, and *Anelloviridae*, together with limited per-sample sequencing depth. Therefore, a vOTU based approach was used for the present dataset. Future studies with greater sequencing depth datasets may benefit from binning-based approaches. Finally, this study did not include strain-level analyses to detect mutations related to antigenic drift, immune escape, or antiviral drug-target variants that could affect field antiviral vaccines or therapies.

## 5 Conclusion

In our study, we profiled the porcine lung virome from 49 lung-derived fluid samples and identified over 18,000 putative operational taxonomic units, with nearly 95% of vOTUs with ≥50% completeness showing no matches in current viral reference databases. Bronchoalveolar lavage samples yielded the highest viral diversity, supporting their use as the optimal method for virome recovery. Predicted viral hosts were consistent with dominant lung bacterial phyla detected in the lung, while auxiliary metabolic genes suggested potential impacts on host metabolic and regulatory processes. Comparative analyses revealed distinct viral families and functions between wild boars and domestic pigs, which may be associated with differences in host ecology and management. Collectively, these results provide one of the first high-resolution catalogs of porcine lung DNA virome and highlight their potential relevance to host-associated microbial ecology and respiratory health.

## Data Availability

The datasets presented in this study can be found in online repositories. This data can be found here: https://zenodo.org/records/20192624.

## Supplementary material

SUPPLEMENTARY FIGURE S1 Sampling distribution and data quality assessment. **(A)** Geographic distribution of sampling sites for domestic pigs and wild boars across China. **(B)** Distributions of raw data size, clean data size, and assembled contig size across all samples. **(C)** Size distribution of raw and clean sequencing data from samples and negative controls. Statistical significance is indicated as follows: ns, not significant; **p* < 0.05; ***p* < 0.01; ****p* < 0.001; *****p* < 0.0001. **(D)** CheckV quality assessment of the 3,565 vOTUs with genome completeness ≥ 50% identified from the pre-filtering dataset of 37,890 vOTUs, showing completeness (%) versus sequence length (log_10_ bp). Colors represent CheckV related quality categories, shapes indicate provirus status, and red numbers denote vOTUs counts within each quality category.

SUPPLEMENTARY FIGURE S2 Assessment of potential contaminant vOTUs and their genomic characteristics. **(A)** Total abundance of contaminant and non-contaminant vOTUs in PBS negative controls and experimental samples. Statistical significance is indicated as follows: ns, not significant; **p* < 0.05; ***p* < 0.01; ****p* < 0.001; *****p* < 0.0001. **(B)** Prevalence distribution of contaminant vOTUs across experimental samples. **(C)** Relationship between GC content and genome size (log_10_-transformed sequence length) of vOTUs. Colors represent viral family assignments.

SUPPLEMENTARY FIGURE S3 Overview of vOTU quality control and community structure. **(A)** Sample-level summary showing the number of vOTUs, average prevalence, and mean RPKM across all samples. Two outlier samples (red) contained fewer than 0.1% of total vOTUs and were excluded from downstream analyses. **(B)** CheckV quality assessment of vOTUs before filtering, showing relationship between genome completeness and sequence length. Colors indicate CheckV quality categories, and shape denote provirus status. Red numeric labels indicate vOTUs counts with each completeness category. **(C)** Hierarchical clustering based on Bray-Curtis dissimilarity calculated from vOTU abundance profiles. Samples cluster according to host origin, separating wild boars and domestic pigs before merging. The heatmap shows pairwise Bray-Curtis distances, and the dendrogram indicates hierarchical relationships among samples. Orange and blue bars indicate wild boars and domestic pigs, respectively.

SUPPLEMENTARY FIGURE S4 Taxonomic annotation of vOTUs from porcine lungs. **(A,B)** Accumulation curves of taxonomic at the Order-level **(A)** and Family-level **(B)** based on the vOTUs dataset, with shaded 95% confidence intervals. **(C–F)** Taxonomic composition of annotated vOTUs across different phylogenetic levels. **(C)** Distribution of vOTUs among viral phyla. **(D)** Family-level composition of all annotated vOTUs. **(E)** Family-level taxonomic composition of vOTUs assigned to *Cressdnaviricota*. **(F)** Family-level taxonomic composition of vOTUs assigned to *Preplasmiviricota*.

SUPPLEMENTARY FIGURE S5 Phylogenetic relationships and genomic characteristics of the 2,559 vOTUs with genome completeness ≥50% (Complete, High-quality and Medium-quality according MIUVIG quality criteria). Proteomic phylogenetic tree constructed from 2,559 vOTUs. From the inner ring to the outer ring, vOTUs are annotated with viral family classification, predicted lifestyle, CheckV quality category, GC content, and genome length (log_2_-transformed).

SUPPLEMENTARY FIGURE S6 Viral lifestyle prediction and host associations in the porcine lung DNA virome. **(A)** Proportions of vOTUs predicted as virulent, temperate, uncertain virulent, and uncertain temperate. **(B)** Distribution of predicted host phyla, shown as the log_10_-transformed counts of vOTUs assigned to each phylum. **(C)** Number of vOTUs across the top 20 predicted host families.

SUPPLEMENTARY FIGURE S7 α-diversity and differential abundance of viral communities between domestic pigs and wild boars across different lifestyle categories. **(A)** Shannon diversity indices of lung DNA viromes between domestic pigs and wild boars across different lifestyle categories, including temperate, uncertain temperate, uncertain virulent, virulent. Statistical significance was determined using the Wilcoxon rank-sum test. Statistical significance is indicated as follows: ns, not significant; **p* < 0.05; ***p* < 0.01; ****p* < 0.001; *****p* < 0.0001. **(B)** Differential abundance of vOTUs based on viral family and lifestyle categories. Each point represents a vOTU with significant differences between domestic pigs and wild boars based on the Wilcoxon rank-sum test, FDR-adjusted *p* < 0.05. The *y*-axis shows the log2 fold change. Enrichment in wild pig samples is shown in the upper region of the plot, while enrichment in domestic pig samples is shown in the lower region. Point colors denote predicted viral lifestyle, and point sizes scale with statistical significance (−log_10_ *p*-values).
