# Supplementary figures and images for: Metagenomic analysis of the DNA virome communities in swine lungs
Source: Front Microbiol. 2026 May 28;17:1798033. doi: 10.3389/fmicb.2026.1798033 (PMC13253880; doi:10.3389/fmicb.2026.1798033)

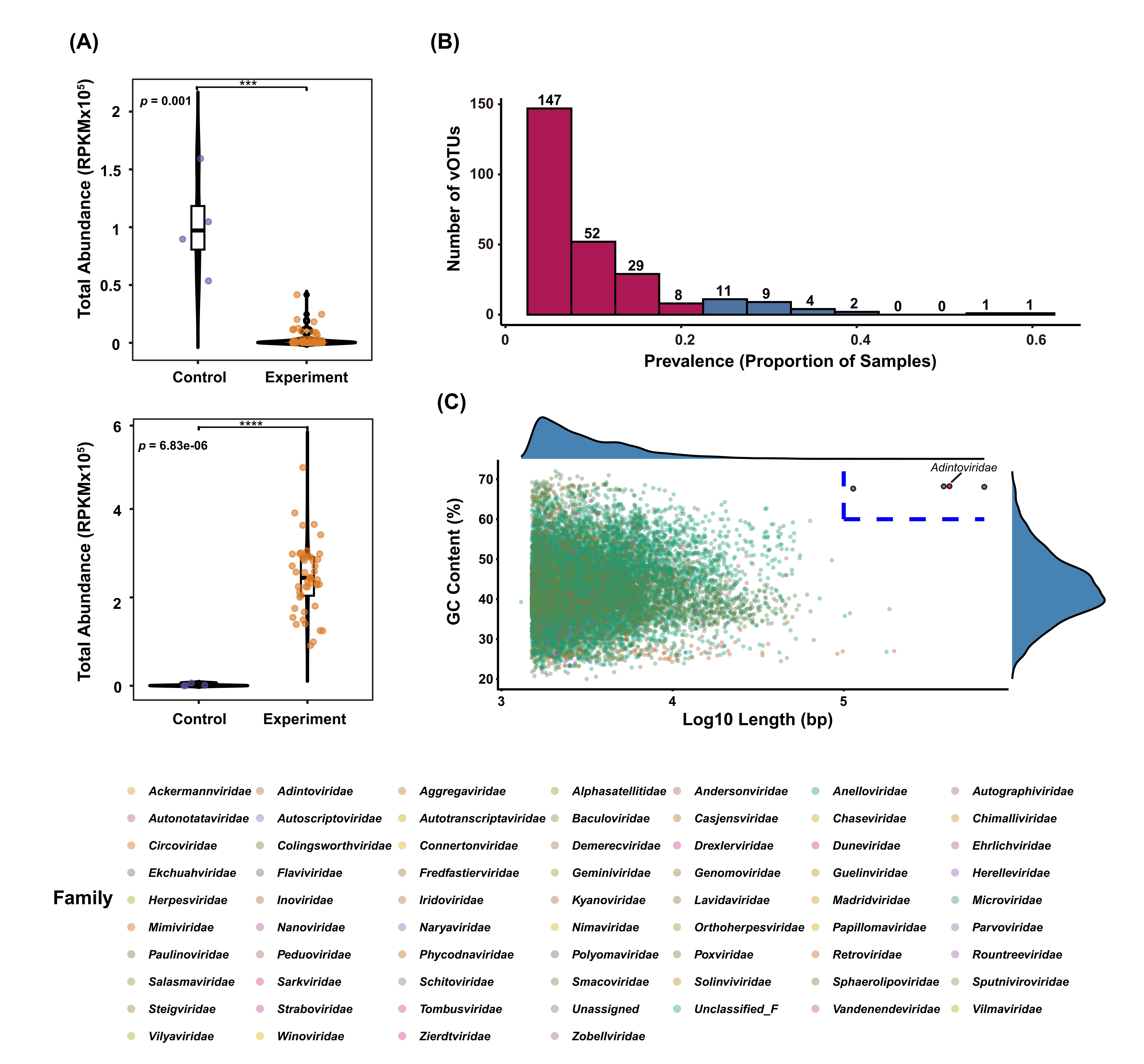

Supplement: SUPPLEMENTARY FIGURE S2 — Assessment of potential contaminant vOTUs and their genomic characteristics. (A) Total abundance of contaminant and non-contaminant vOTUs in PBS negative controls and experimental samples. Statistical significance is indicated as follows: ns, not significant; *p < 0.05; **p < 0.01; ***p < 0.001; ****p < 0.0001. (B) Prevalence distribution of contaminant vOTUs across experimental samples. (C) Relationship between GC content and genome size (log10-transformed sequence length) of vOTUs. Colors represent viral family assignments. [file Image_2.JPEG]

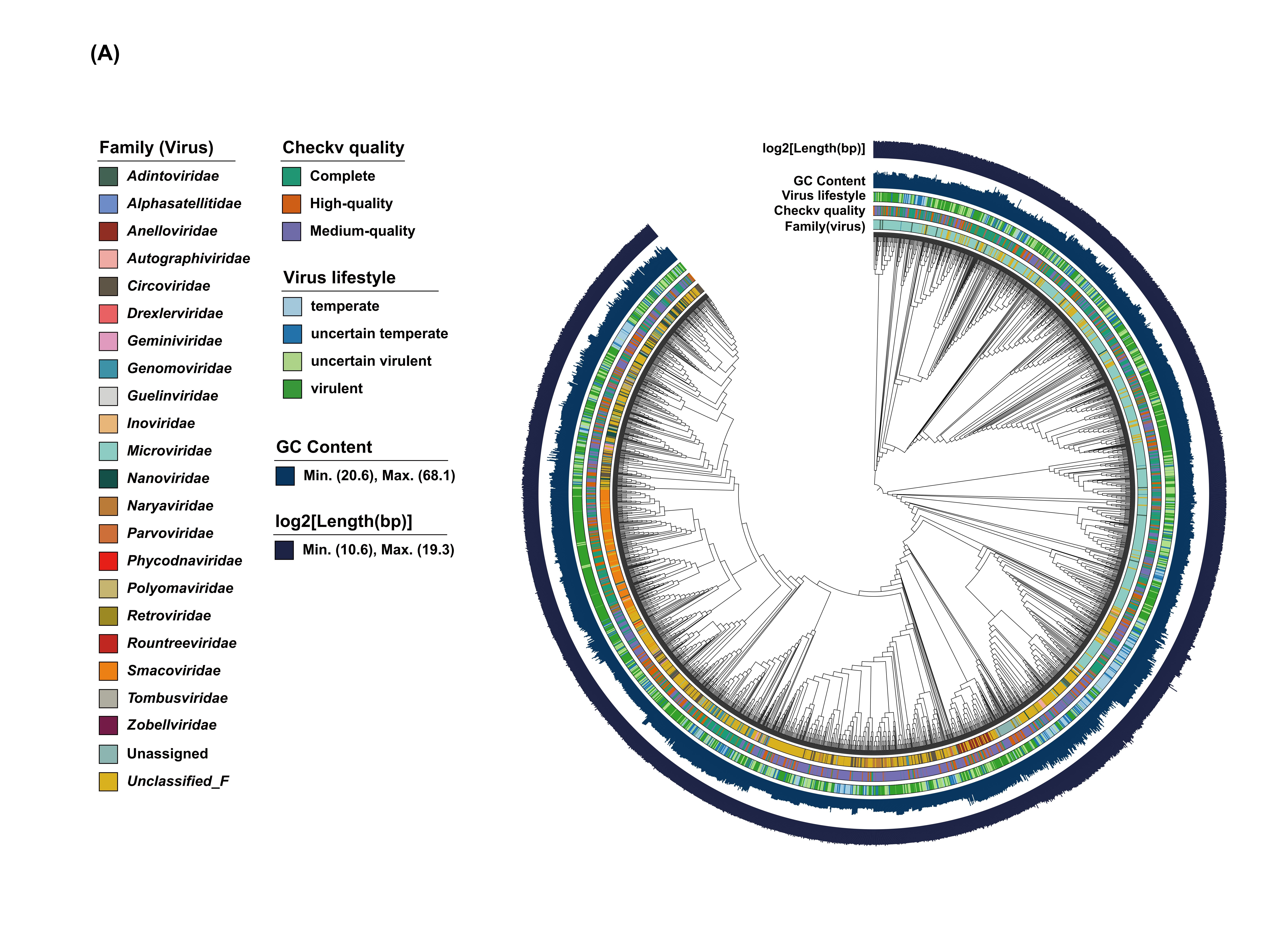

Supplement: SUPPLEMENTARY FIGURE S5 — Phylogenetic relationships and genomic characteristics of the 2,559 vOTUs with genome completeness ≥50% (Complete, High-quality and Medium-quality according MIUVIG quality criteria). Proteomic phylogenetic tree constructed from 2,559 vOTUs. From the inner ring to the outer ring, vOTUs are annotated with viral family classification, predicted lifestyle, CheckV quality category, GC content, and genome length (log2-transformed). [file Image_5.JPEG]

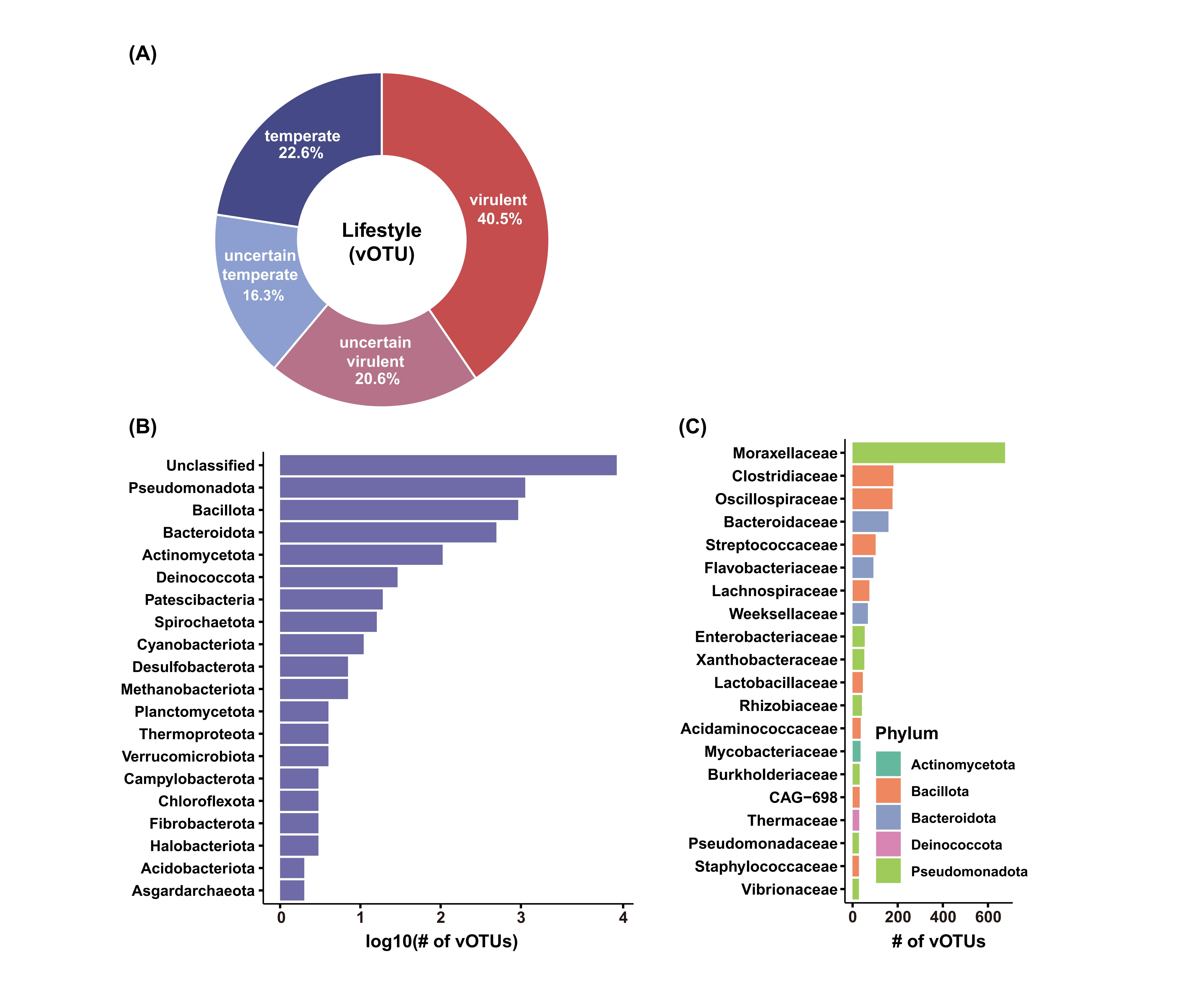

Supplement: SUPPLEMENTARY FIGURE S6 — Viral lifestyle prediction and host associations in the porcine lung DNA virome. (A) Proportions of vOTUs predicted as virulent, temperate, uncertain virulent, and uncertain temperate. (B) Distribution of predicted host phyla, shown as the log10-transformed counts of vOTUs assigned to each phylum. (C) Number of vOTUs across the top 20 predicted host families. [file Image_6.JPEG]
